# Tunable dislocations overcome mechano-functional tradeoff in perovskite oxides

**DOI:** 10.1126/sciadv.aed0057

**Published:** 2026-08-07

**Authors:** Jiawen Zhang, Wenjun Lu, Xufei Fang

**Affiliations:** ^1^ Department of Mechanical and Energy Engineering, Southern University of Science and Technology, Shenzhen, 518055, China.; ^2^ Institute for Applied Materials, Karlsruhe Institute of Technology, Karlsruhe 76131, Germany.

## Abstract

Recent advancements in dislocation engineering are reshaping the traditional view towards ceramics being brittle. Here, we use KTaO_3_ (KTO), a perovskite oxide that is newly discovered with room-temperature bulk plasticity, and demonstrate that the seeded dislocations can effectively tune both mechanical and functional properties. We uncover a brittle-ductile-brittle (BDB) transition: low dislocation densities lead to brittle failure, intermediate densities (∼10^14^ m^−2^) enable superior compression plastic deformation capacity with strains over 20%, and high dislocation densities (∼10^15^ m^−2^) induce brittle fracture again. This dislocation density-dependent non-monotonic mechanical response challenges the traditional behavior of ceramics and offers design opportunities. Furthermore, dislocation densities can monotonically decrease thermal conductivity, revealing a tradeoff between mechanical strength and functionality. The findings reveal a critical threshold of dislocation density in optimizing the performance of functional oxides, and provide a framework for using dislocations to design advanced materials where mechanical durability and enhanced functionality are intertwined.

## Teaser

Tunable room-temperature dislocations in KTaO_3_ reveal a brittle-ductile-brittle transition and modulated functionality.

## INTRODUCTION

Ceramics are typically categorized as brittle materials, exhibiting negligible plastic deformation under ambient conditions. This perception, long embedded in both scientific understanding and industrial design, stems from the strong and directional ionic/covalent bonding that causes high lattice friction stress and insufficient operative slip systems at room temperature (*1*). The direct consequence is a lack of easy dislocation (1D crystalline defect) glide in ceramics, as compared to metallic materials (*1*). Up to date, the potential of ceramics has been shackled to passive structural applications, where strength and hardness are prioritized over plastic deformation and toughness (*2*) despite the fact that the latter has been pursued continuously (*3*, *4*). Meanwhile, tuning the functional properties of ceramics has been limited to 0D point defect engineering via defect chemistry (*5*, *6*) or 2D boundary engineering through interface design (*7*) or texturing (*8*, *9*). Dislocation as a fundamental defect type has unfortunately long been cast out for ceramics engineering. However, recent breakthroughs in dislocation engineering in oxides (*10*), particularly dislocation seeding (*11*) involving direct mechanical imprinting into ceramic oxides, have begun to fundamentally challenge this conventional view and open frontiers in the mechanical and functional design of oxides (*12*, *13*), with a potential to be extended to more than dozens of ductile ceramics that exhibit room-temperature bulk plasticity mediated by dislocations (*14*).

Perovskite oxides, because of their rich electronic properties and structural tunability, have become archetypal platforms for exploring dislocation-based novel properties (*12*, *15*–*18*). Among them, SrTiO_3_ (STO) and KTaO_3_ (KTO), as prototypical functional oxides, have attracted particular attention for their capacity to host dense dislocation networks by bulk compression at room temperature (*19*–*23*). These materials not only exhibit tunable electronic and electromechanical responses to dislocations, but also serve as model systems to investigate room-temperature bulk plasticity in crystalline oxides from a fundamental perspective. For instance, the current authors have recently demonstrated that STO can be mechanically conditioned to exhibit large room-temperature plasticity with more than 30% plastic strain via a strategy with controlled dislocation seeding (*11*) to overcome dislocation nucleation, a common bottleneck for achieving plasticity in ceramics. By incrementally modulating dislocation densities ranging from ∼10^10^ to 10^14^ m^−2^ in single-crystal STO, this enables systematic investigations of the relationship between dislocation density and mechanical response in a structurally cleaner system (*24*).

**Fig. 1.**
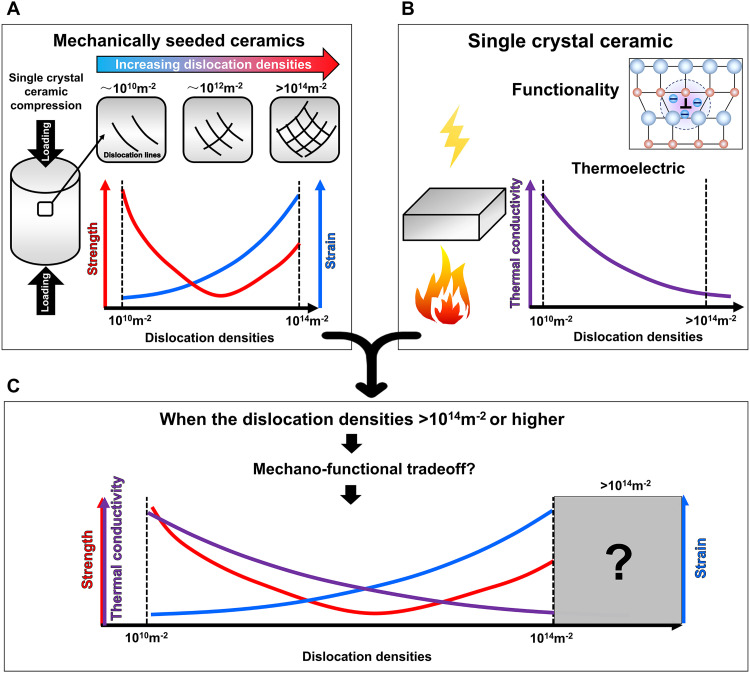
Mechanically seeded dislocations in brittle functional single-crystal ceramic materials. (**A**) Strength and strain as a function of the tunable dislocation densities in functional ceramics [with a maximum density of ∼10^14^ m^−2^ as state of the art (*24*)]; (**B**) Thermal conductivity as a function of dislocation density; (**C**) Beyond the limit: what changes in mechanical properties would occur if the dislocation density surpasses the limit of ∼10^14^ m^−2^? Note the mechano-functional properties tradeoff as a function of dislocation density.

A key finding from this line of research was the emergence of the non-monotonic mechanical behavior: the yield strength of STO initially decreased as dislocations were introduced, attributed to enhanced mobility and dislocation glide, but then the yield strength increased with higher dislocation densities due to forest hardening and dislocation-dislocation interactions (*24*–*26*). This “V-shape” behavior in strength (Fig. 1A), reminiscent of dislocation nucleation, multiplication, and work-hardening phenomena in metals (*27*), marked a conceptual breakthrough in ceramics as it challenged the brittle paradigm of oxide ceramics and provided a fundamental pathway toward tailoring plastic deformation through tunable dislocation densities (*10*, *24*). However, the state-of-the-art dislocation density plateaued in STO around 10^14^ m^−2^, beyond which further densification was hindered by brittle fracture, limiting access to the full mechanical landscape. This research displays a distinct dislocation behavior such as the much higher capacity in dislocation multiplication and higher degree of freedom for dislocation density tuning in KTO compared to STO. We extend the partial picture of a one-way brittle-to-ductile transition in STO to a fully brittle-to-ductile-to-brittle (BDB) transition in KTO. This has been challenging since achieving extremely high dislocation density up to ∼10^15^ m^−2^ without cracking the samples is not a given in perovskite oxides.

Equally important, the mechanics-enabled dislocation engineering approach has catalyzed the assessment of dislocation-tuned functional properties (*28*). Driven by the new research horizon of dislocation technology in functional oxides (*10*, *13*, *28*, *29*), one rising research trend has been centered on pushing the limit of dislocation density in ceramics to maximize the physical properties, for instance, boosting electromechanical properties in ferroelectrics (*12*), enhancing the electrocatalytic performance by way of dislocation-modified hydrogen adsorption free energy (*30*), increasing electrical conductivity by overlapping the space charge zones around charged dislocations (cores) (*31*, *32*), and decreasing thermal conductivity due to effective phonon scattering (*33*), with the latter two combined to boost thermoelectric figure of merit for energy harvesting (*34*, *35*).

In addition to the first finding of dislocation-regulated BDB in perovskite oxide, we note this is one of the first reports to address the simultaneous impact of dislocation-tuned mechanical and functional properties. A consensus appears to be that the higher the dislocation density, the better the outcome of these functional properties, reflected by a monotonic correlation between them, as schematically represented for thermal conductivity in Fig. 1B. Yet up to date, these two pieces of the puzzle concerning the mechanical and functional aspects have not been put together due to these two much divided research fields. When fusing these two aspects (Fig. 1C), namely, non-monotonic change in strength but monotonic variation in physical properties as a function of increasing dislocation density, clearly a tradeoff needs to be sought. In this study, we define a “tradeoff” as the existence of a competing relationship where the dislocation density that optimizes one set of deformation ability and strength unavoidably shifts thermal conductivity for other applications. This tradeoff will be crucial for the integration of mechanical integrity and functional performance for designing next-generation dislocation-based devices.

To establish a model case, here we used KTO as a new prototypical perovskite oxide (fig. S1) alongside STO to assess the dislocation-tuned functional properties in combination with the mechanical properties. KTO has recently been discovered to exhibit large room-temperature bulk plasticity by the current authors (*21*) in parallel with Greven’s group (*22*). A new set of bulk compression test for KTO is also showcased in fig. S2. Analogous to STO, KTO is cubic at room temperature and is one of the most widely studied perovskite oxides that has received extensive research in recent years for its structural and functional versatility (*21*–*23*, *36*, *37*). Nevertheless, compared to STO, distinct structural and electronic properties exist in KTO. The latter possesses a larger lattice parameter, enhanced chemical stability, and a more covalent Ta-O bonding character, all of which influence its dislocation behavior differently than STO (*22*, *23*). KTO is also used to address the generality of the dislocation seeding approach recently validated in STO (*11*). The underlying mechanisms are yet to be explored by the community, but since KTO is one of the most important perovskite oxides for versatile functional applications (*36*, *38*), we report this model material here in this work.

## RESULTS

### Ex situ micropillar compression tests

**Fig. 2.**
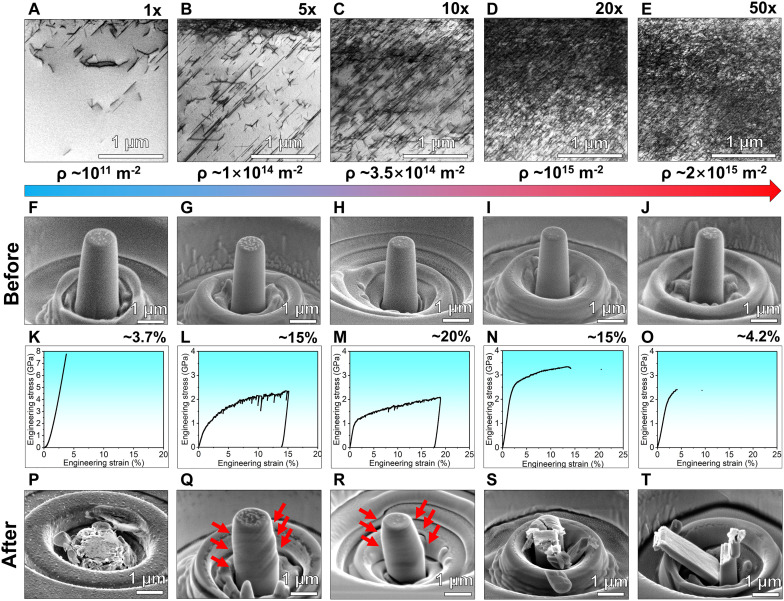
Dislocation density tuning and micropillar compression tests in dislocation-seeded samples. Dislocation density tuning is achieved by using a Brinell indenter for scratching the sample surface with different passes (i.e., via cyclic scratching, details in Materials and Methods). (**A** to **E**) Annular bright field (ABF)-scanning transmission electron microscopy (STEM) images showing a progressive increase in dislocation density beneath the scratch area with increasing number of scratch passes (1× to 50×, note 1× means 1 pass), ranging from ∼10^11^ to ∼10^15^ m^−2^. (**F** to **J**) Scanning electron microscopy (SEM) images of micropillars with varying seeded dislocation densities before ex situ compression, which eliminates the electron beam effect. (**K** to **O**) Engineering stress–strain curves of the deformed micropillars. (**P** to **T**) SEM images of micropillars after compression, with arrows in (Q) and (R) indicating the slip traces for dislocation activity, while those with much lower dislocation density in (P) shows completely brittle shatter, and these with ultra-dense dislocations in (S) and (T) display again crack and splitting in a brittle manner.

As illustrated in Fig. 2A, the high tunability of dislocation densities in KTO enabled a remarkably extended regime beyond ∼10^15^ m^−2^ [at least one order of magnitude higher than that in single-crystal STO (*24*)]. This unprecedentedly high value reveals additional features to the non-monotonic mechanical response: as the dislocation density increases, the material goes through a brittle-ductile-brittle transition, as evidenced by the micropillar compression. With a low dislocation density (Fig. 2, A, F, K, and P), brittle fracture ensues right after the elastic deformation, as evidenced by the abrupt failure indicated in the stress-strain curve and the completely crushed pillar after deformation.

As the dislocation density increases, a notable shift occurs: the stress-strain curves exhibit smooth yield and clear work hardening behavior, with total strains exceeding ∼15% (Fig. 2, B, G, L, and Q) and ∼20% (Fig. 2, C, H, M, and R; and fig. S3A), indicative of stable dislocation glide and strain accommodation. Quantitative statistical analysis of intermittent stress drops in KTO stress-strain curves as a function of dislocation density was shown in fig. S4. The average stress drop decreases from approximately 125 MPa (5×) to 55 MPa (10×), then to 15 MPa (20×) as the dislocation density increases (fig. S4D). This is consistent with a transition from localized dislocation avalanche behavior to more uniform dislocation flow (*39*).

When the dislocation density exceeds a critical threshold (∼10^15^ m^−2^), brittle fracture re-emerged (Fig. 2, N, S, O, and T; and fig. S3B). We note that the 20× scratched and ∼1 μm diameter micropillars (Fig. 2D) exhibited a gradient dislocation distribution, whereas the other samples displayed a uniform distribution with a height of ∼3 μm. The impact of such a gradient structure on the mechanical properties, particularly on the detailed BDB behavior, remains an open question and will be investigated in the future. It will be worth investigating the gradient effect by designing different gradients to address this gradient effect in the future. This dislocation density dependent BDB phenomenon has not been reported before for oxide ceramics. The fracture/yield strengths exhibit a similar but more pronounced non-monotonic change as a function of the dislocation density, as summarized in Table S1. Both mechanical responses (strength and strain) suggest that excessively high dislocation density destabilizes plastic flow and increase the chance for cracks to occur. It is worth emphasizing that even when the dislocation density in KTO reaches ∼10^15^ m^−2^, the crystal structure remains single-crystalline, without undergoing polycrystallization or amorphization (fig. S5). This remarkable structural stability highlights the intrinsic resistance of KTO against defect-induced structural degradation, underscoring a fundamental distinction between complex oxides and conventional metallic systems. In addition, as shown in fig. S6, increasing the pillar size up to approximately 3 μm leads to noticeable changes in the yield strength of the samples, while the overall deformation mechanisms remain essentially unaffected. This observation suggests that, although larger dimensions can influence the onset of plasticity (*40*, *41*), the fundamental deformation behavior of the material is size-independent within this range. The underlying mechanisms will be discussed later based on the dislocation pileup and work hardening.

### In situ nanopillar compression tests

**Fig. 3.**
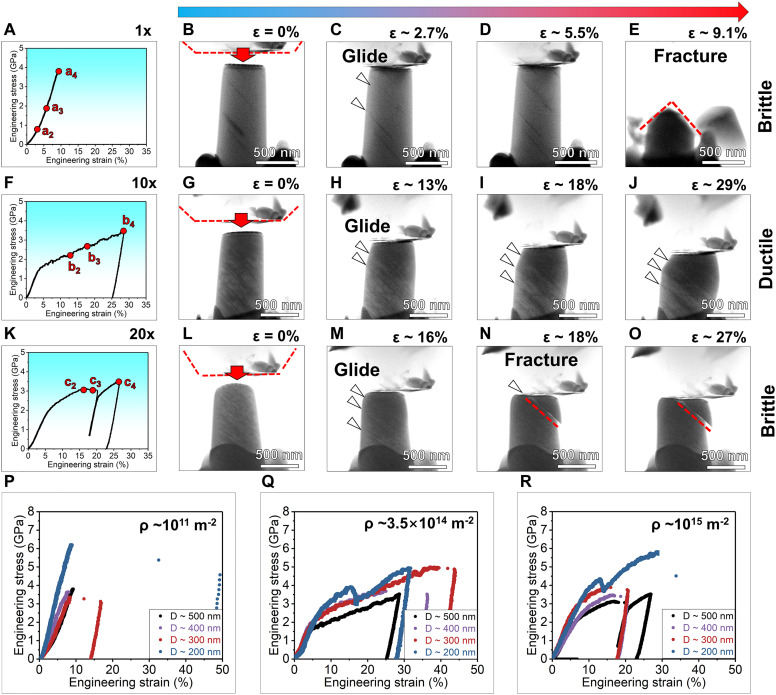
In situ nanopillar compression in ABF-STEM imaging mode. (**A**) Engineering stress-strain curve for sample with 1× scratch, (**B** to **E**) corresponding image snapshots from the in situ test, where fracture occurs at a maximum strain of ∼9%. (**F**) Stress-strain curve of the nanopillars in the 10× scratched sample, (**G** to **J**) corresponding image snapshots visualizing numerous slip bands (indicated by arrows), with the nanopillar achieving up to ∼30% strain without fracture. (**K**) Stress–strain curve of the 20× scratched sample, (**L** to **O**) corresponding image snapshots reveal fracture occurs at a maximum strain of ∼18%. (**P**) Stress-strain curves of pristine samples with different nanopillar sizes, all exhibiting brittle fracture. (**Q**) Stress-strain curves of 10× scratched samples with varying pillar sizes, all displaying stable plastic deformation beyond ∼30% strain. (**R**) Stress-strain curves of 20× scratched samples with varying pillar sizes, showing a combination of plastic deformation and fracture, and an overall reduced plasticity compared to the 10× scratched case. The movies can be found in Supplementary Materials/Movies.

To directly visualize the deformation processes and shed light on the mechanisms governing the BDB transition in KTO, we further conducted in situ nanopillar compression experiments. To highlight the dislocation structure within the pillars, the brightness and contrast of the STEM images in Fig. 3 have been adjusted, and the indenter position has been marked with red dashed lines in Fig. 3, B, G, and L. Representative engineering stress-strain curves (Fig. 3, A, F, and K) and corresponding snapshots confirm the influence of dislocation density on the various deformation responses. The pristine sample (1× scratched, Fig. 3, A to E) exhibits classic brittle fracture, with no evidence of plastic yield and limited dislocation activity prior to the catastrophic brittle failure (Fig. 3E). In contrast, the 10× scratched sample (Fig. 3, F to J) demonstrates remarkable plasticity, sustaining over 30% compressive strain without cracking. The snapshots from the in situ tests (Fig. 3, G to J) capture the continuous formation and propagation of dense slip bands, signifying profuse dislocation multiplication and strain delocalization. This plastic deformation regime, enabled by an intermediate dislocation density (∼10^14^ m^−2^), aligns with the observations in micropillar tests. Continued increase in the dislocation density (20× scratched sample, Fig. 3, K to O) leads to an early onset of fracture at a total strain of ∼18%. Although dislocation slip is still visible (Fig. 3, L to O), the deformation becomes less homogeneous, with localized shear preceding failure. These findings confirm the non-monotonic mechanical response as a function of dislocation density: an initial enhancement of plasticity via mobile dislocation networks is followed by a re-emergence of brittleness due to dislocation pileup and dynamic jamming.

To further address the size effect in nano−/micropillar compression, as most widely discussed in metals (*40*, *41*), we perform systematic size-dependent tests (Fig. 3, P to R and figs. S7 to S10) to confirm that this BDB transition persists across varying pillar diameters. Summary data were plotted in fig. S11. Since the taper angles slightly increased with the decrease of the pillar size, we employed the corrected equation $D_{a}$ = $D_{t}$ + Htanα to calculate the stress value in Fig. 3 (A, F, and K), where $D_{a}$ is the top diameter, H is the height of the micropillar (*42*, *43*). The results suggest that this dislocation density dependent BDB behavior is an intrinsic feature induced by dislocations and not limited by dimensional constraints or the pillar size. These real-time in situ observations in nanopillars (Fig. 3) align with the ex situ micropillar compression tests (Fig. 2) and confirm the role of dislocation-mediated mechanisms in tuning plasticity in KTO, highlighting the critical dislocation density window in which optimal mechanical properties can be achieved. The extremely high-density dislocations do not always merit a better mechanical performance.

### Dislocation core characterization of KTO

**Fig. 4.**
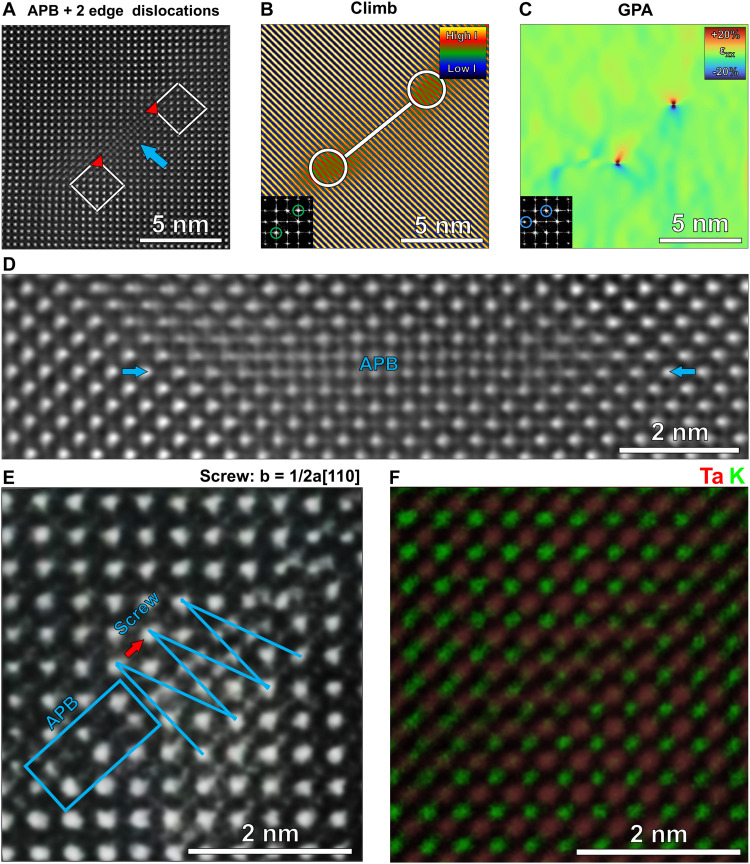
Atomic-resolution HAADF-STEM and EDS mapping of the 10× scratched KTO crystal. (**A**) Atomic-resolution HAADF-STEM image of KTO along the <001> zone axis, illustrating an anti-phase boundary (APB), as indicated by the blue arrow. The APB is terminated by two edge dislocations in climb dissociation configuration with Burgers vectors of 1/2 <110>. (**B**) Inverse fast Fourier transform (IFFT) image highlighting the positions of the two edge dislocations. (**C**) GPA strain map corresponding to panel (A), visualizing the strain distribution associated with the edge dislocations. (**D**) Magnified view of the APB structure from panel (A), displaying an atomic arrangement that differs from conventional edge or screw dislocations. (**E**) Atomic-resolution HAADF-STEM imaging and (**F**) EDS mapping of both the screw dislocation and the APB region, highlighting local compositional variations.

To identify the nature of the dislocations introduced in the 10× scratched KTO sample (the intermediate density in this condition merits its selection), we first employed two-beam conditions TEM analysis. This technique allowed us to unambiguously confirm the presence of both edge and screw dislocations, as illustrated in fig. S12. Such direct observation is key for further analysis of how these distinct dislocation types contribute to the observed mechanical responses. To elucidate the atomic-scale origin of the ductile regime observed in KTO, we performed high-resolution high-angle annular dark-field (HAADF)-STEM and energy dispersive spectroscopy (EDS) analyses on the 10× scratched sample, which exhibited the peak performance in plasticity. Fig. 4A reveals a distinct antiphase boundary (APB) within the lattice, terminating at two edge dislocations in a climb dissociation configuration with ½<110> Burgers vectors (Fig. 4B). This configuration evidences a strong interaction between dislocations and planar defects, suggesting that APBs may serve as barriers for dislocation motion (*44*). Strain mapping via geometric phase analysis (GPA, Fig. 4C) reveals pronounced local lattice distortion in the vicinity of the dislocation cores, confirming their strain-bearing nature. Notably, the APB structure (Fig. 4D) displays an ordered atomic configuration distinct from classical dislocation lines, implying a unique core structure possibly stabilized by ionic mismatch or charge redistribution (*23*). HAADF-STEM imaging combined with elemental mapping (Fig. 4, E to F) further reveals localized compositional fluctuations at the screw dislocation-APB interface, particularly in the distributions of K and Ta. Such heterogeneity can effectively modulate local bonding strength and defect energetics, facilitating dislocation nucleation and/or pinning. These results point to a cooperative mechanism in which dislocation-APB interactions mediate plastic flow, enhancing compression plastic deformation capacity at moderate defect densities.

More interestingly, comparative analysis with STO (*24*) reveals that KTO possesses a greater propensity for forming complex dislocation-APB networks, consistent with its enhanced capacity for dislocation multiplication (with a much higher dislocation density under the same mechanical loading conditions) and higher threshold of fracture strain. Compared to STO, KTO exhibits a lower APB formation energy. The APB formation energy is 0.925 J m^−2^ and 0.280 J m^−2^ for STO and KTO, respectively (*23*, *45*). This structural flexibility may arise from the more covalent Ta–O bonding and less rigid octahedral framework in KTO, allowing for easier accommodation of lattice distortions, based on the most recent molecular dynamics simulations (*23*). Together, these atomic-resolution insights indicate that the BDB transition in KTO appears more than merely a function of dislocation density, but intricately governed by the structural change and chemical fluctuation of dislocation-defect interactions, providing a mechanistic basis for defect-informed design of deformable ceramics.

### Thermal conductivity measurements

**Fig. 5.**
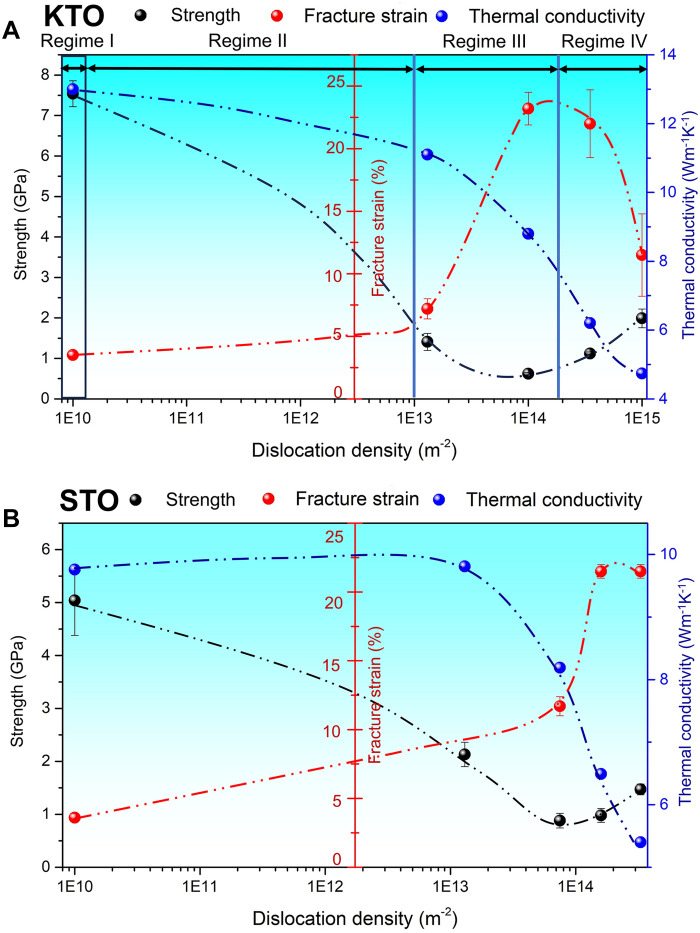
An overview of how dislocation density simultaneously governs mechanical response and thermal conductivity in KTO and STO, revealing monotonic and non-monotonic trends. (**A**) Plot showing the variation of the mechanical behavior: fracture (Regime I), stochastic fracture or yield (Regime II), plastic yield (Regime III and IV), and fracture strain in micropillar compression, as well as thermal conductivity as a function of dislocation density for the KTO crystal. Thermal conductivity decreases monotonically with increasing dislocation density, while mechanical properties exhibit a non-monotonic evolution, i.e., transitioning from brittle fracture at low densities to enhanced plasticity at intermediate levels, before degrading again at high densities. Note the four different regimes I, II, III, and IV for indications. (**B**) Similar plot as in (A) for STO crystal. With mechanical data reproduced from recent literature (*24*), and the thermal conductivity measured in this work.

Next to the mechanical properties, we further investigate the functional properties tuned by dislocations by showcasing the thermal conductivity (see Materials and Methods). Fig. 5A compiles the yield strength, fracture strain, and thermal conductivity for KTO as a function of increasing dislocation density. This more complete picture reveals two interesting features. First, the thermal conductivity decreases monotonically as the dislocation density increases. This is expected as the large strain field around dislocations has long been proposed (*46*–*48*) and recently evidenced by room-temperature observation to effectively scatter phonons to reduce thermal conductivity (*33*). Note that the slope becomes steeper with the increased dislocation density above ∼10^13^ m^−2^. This can be attributed to the fact that, in addition to the dislocations as line defects for phonon scattering, the increased densities in APBs as 2D defects (Fig. 4) will add to the phonon scattering as the dislocation density increases. The contribution of boundary scattering has been extensively modeled and validated elsewhere (*35*, *49*). Second, the mechanical responses exhibit a clear non-monotonic change. In the samples with low dislocation densities (<10^13^ m^−2^), brittle fracture with high fracture strength (>7 GPa) and negligible plastic strain (<1%) prevails. As dislocation density increases, the capability to plastically deform improves markedly, reaching a maximum at ∼10^14^ m^−2^, where plastic strains approaching 25% without catastrophic failure. Beyond this threshold, the trend in plastic deformability starts to reverse, with a fracture strain declining sharply.

For the sake of generality, in Fig. 5B we present the data for STO, with an analogous behavior observed. This demonstrates that such dislocation density-dependent competition between the mechanical and functional properties is not unique to KTO, but should hold true for other perovskite oxides provided that dislocations can be engineered. Nevertheless, KTO exhibits a more dramatic change especially in the fracture strain compared to STO, due to the fact that a one order of magnitude higher dislocation density is achievable in KTO.

## DISCUSSION

### BDB transition

The underlying mechanism for the V-shaped curve for the strength as a function of dislocation in STO (*11*) has been interpreted within the framework of dislocation mechanics similar to metals by El-Awady *et al.* (*27*). Here, we focus on the BDB transition in KTO at room temperature, where the brittle fracture at both ends of the curve at both low (∼10^12^ m^−2^) and extremely high (∼10^15^ m^−2^) dislocation densities features a distinction in ceramics compared to metals, while the most significant plastic strain is achieved at moderate dislocation density (∼10^12^ m^−2^ to ∼10^14^ m^−2^). While the BDB transition has now been firmly established in KTO, whether this behavior generalizes to other ceramic systems remains an open question. We hypothesize that such a BDB transition should occur in ceramics where dislocation mobility is readily available; meanwhile, the dislocation density can be controlled over a wide range. Direct experimental validation in other systems (*14*) is required to verify this hypothesis in the future.

In metals, particularly BCC metals such as tungsten, the brittle-ductile transition is controlled by dislocation mobility as function of temperature (*50*). This is not the case in ceramics as their plasticity is restricted rather by dislocation nucleation (*11*, *51*). This shift of the bottleneck is caused by the fundamentally different processing routes for forging metals and sintering ceramics. Conventionally sintered ceramics by high-temperature firing or grown crystals exhibit approximately 4–5 orders of magnitude lower dislocation densities compared to metals. Down to the nano−/microscale, there is barely one dislocation existing in the deformation volume in ceramics (Regime I, fracture, Fig. 5A). Hence, for plasticity to occur it requires dislocation nucleation at a stress level approaching a fraction of the shear modulus to break the bonds in shear deformation (*52*, *53*). This picture has been fundamentally changed by dislocation seeding to circumvent the high nucleation barrier and shift the restricting process to dislocation motion and multiplication, which proves much easier in dozens of ceramics (*10*, *11*, *14*). In the stochastic transition Regime II, stochastic fracture or plastic deformation was correlated with the number of defect sources. In this regime, pillars show rare dislocation nucleation and events in a random manner but fail to propagate or multiply due to rapid exhaustion, resulting in intermediate stochastic plasticity (*54*). In particular, dislocation motion and multiplication are operating more effectively to carry the plasticity in the intermediate range (Regime III, plastic deformation, Fig. 5A) for both representative perovskite oxides KTO and STO (∼10^12^–10^14^ m^−2^). However, at ultrahigh densities (Regime IV, fracture, Fig. 5A), dislocation-dislocation interactions and dislocation forest hardening, as well as entanglement with APBs in the case of KTO, start to dominate. The much-limited available slip systems in perovskite oxides at room temperature [6 physically distinct but only 2 independent slip systems in STO and KTO with the cubic structure (*1*)] and limited ability to cross slip due to the dissociation of dislocations at ambient conditions lead to more severe dislocation jamming to nucleate cracks (*24*, *55*–*58*). In short, the dynamics between available mobile dislocations and immobile pinning structures (being either sessile dislocations or APBs) likely governs the observed BDB transition in KTO. It is worth noting that the present study is focused on the (001) orientation. The influence of different orientations [e.g., (011) and (111)] as well as the dislocation density on the BDB behavior in KTO is of interest, if crystallographic orientations are considered. Given the fact that KTO and STO are similar in dislocation mechanics, the previous investigation on the plasticity in STO with (001), (011), and (111) surfaces at room temperature is worth being referred to the future experimental design (*14*).

### Size effect

Here, we use the unified parameter D*$\sqrt{\rho }$ to compare the change of fracture strain and yield strength with the sample’s characteristic length D (pillars’ diameter), as displayed in fig. S14 and table S2. With D*$\sqrt{\rho }$ < 1, the micropillars typically exhibit brittle fracture, accompanied by high strength. As dislocation density increases, the deformation behavior transforms into a stochastic transition regime, and the pillar is in a state between fracture and the point where plastic deformation can begin to occur when 1 < D*$\sqrt{\rho }$ < 3. As the value D*$\sqrt{\rho }$ is over 3, plastic deformation is dominated by dislocation multiplication and motion. However, with dislocation density beyond some critical value N_c_ (e.g., D*$\sqrt{\rho }$ ≥ N_c_), strong dislocation pileup may induce cracking again. As shown in the calculated results in Table S2 in our study, this critical value N_c_ is approximately 50–60, which means that when N_c_ is over this value, the plastic deformation capacity of the pillar will decrease.

Both tests at nanoscale and microscale, being in situ or ex situ, validates that the density-mediated properties are consistent at small scales. Since both KTO and STO are experimentally demonstrated to be capable of room-temperature bulk plasticity (*20*–*22*), and the dislocation seeding approach using Brinell indentation and scratching to generate meso/macroscale plastic zone size applies (*21*, *59*), it is in principle feasible to scale it up to meso/macroscale for the density-dependent measurement. Nevertheless, consider the high cost and limited availability of large single crystals of such perovskite oxides, one of the most promising application scenarios shall be at the small scale for potential MEMS/NEMS devices using dislocations, matching the length scales investigated here.

### Mechano-functional tradeoff

The finding of the dislocation density-dependent BDB transition deserves attention for the endeavors in dislocation-tuned functionality of ceramics, for which ultrahigh dislocation density is desirable. Next to the finding of dislocation-regulated BDB in perovskite oxide, we note this is one of the first reports addressing simultaneously the competing impact between dislocation-tuned mechanical properties and functional properties, except for the most recent research on ferroelastic polycrystalline oxide that addresses the tradeoff relationship between the fracture toughness and thermal conductivity via high density dislocations achieved through processing (*60*). Here we mainly demonstrate the thermal conductivity as a monotonic function of dislocation density over the whole achievable range, for both STO and KTO. Nevertheless, dislocation-tuned electrical conductivity has been reported to exhibit analogous monotonic behavior (*12*, *18*, *31*, *61*). By controlling dislocations, it is possible to achieve multi-functional properties alongside reliable mechanical properties, for example, attaining both low thermal conductivity and high electrical conductivity simultaneously (fig. S16). Given the scenarios, next to the superior functional performance, where excellent mechanical integrity and structural robustness of the components is critical, the tradeoff between the mechanical and functional properties, namely, mechano-functional tradeoff mediated by dislocations in ceramics shall be borne in mind. This is relevant for designing emerging functional dislocation-based devices operating in multifield-coupled and particularly mechanical load-bearing environments.

In conclusion, this work provides a framework for dislocation engineering in plastically deformable functional oxides. The direct comparison between dislocation-tuned mechanical and functional properties shall trigger a reassessment of the role of dislocations in these materials, especially in the extremely high dislocation density regime (∼10^15^ m^−2^). By demonstrating a controllable dislocation-density-based BDB transition in perovskite SrTiO_3_ and KTaO_3_, we demonstrate that compression plastic deformation in functional oxides is not a binary state but rather a dynamic outcome governed by defect topology, density, and interaction mechanisms. This BDB transition, distinct from metallic systems and any other ductile ceramics reported before, reveals a fundamental limit in dislocation-mediated toughening: while defect engineering can enhance plasticity, there exists a critical density beyond which the crystal loses its ability to accommodate strain plastically. Understanding this tipping point is essential for optimizing mechanical robustness in oxide-based devices, particularly those involving mechanical/multi-field loading. Our findings advance the defect-mechanics-functionality paradigm beyond traditional endeavor in ceramics and open avenues for engineering functional oxides.

## MATERIALS AND METHODS

### Mechanical seeding of dislocations

The (001)-oriented KTaO_3_ single crystals were acquired from Hefei Ruijing Optoelectronics Technology Co., Ltd. (Anhui, China). The KTaO_3_ thin plates with a dimension of 5 mm by 5 mm by 1 mm were single-side polished to a surface roughness lower than 2 nm. A friction and wear machine (RTec, MFT) equipped with a 3 mm diameter α-Al_2_O_3_ ball was employed to generate different wear tracks by tuning the cyclic scratching passes (fig. S13). The scratching load was set to 8 N, with a scratching speed of 0.2 mm/s and distances of 2 mm. The optimized parameters ensure no surface cracks were generated.

### TEM analysis

The TEM specimens were lifted out along the scratching direction in the center of the wear tracks using a dual-beam focused ion beam (FIB) in an SEM (Helios Nanolab 600i, FEI, Hillsboro, USA). ABF-STEM images were captured using a TEM instrument (FEI Talos F200X G2, Thermo Fisher Scientific, USA) operating at 200 kV. A probe semi-convergence angle of 10.5 mrad and inner and outer semi-collection angles of 12–20 mrad were used. For atomic-scale imaging, HAADF-STEM imaging was then performed in a double aberration-corrected transmission electron microscope (Titan Themis G2, FEI, Netherlands) operated at 300 kV. A probe semi-convergence angle of 17 mrad was utilized, with inner and outer semi-collection angles ranging from 38 to 200 mrad.

### Pillar compression

For micropillar compression tests, all the micropillars were prepared in the FIB with decreased ionic current of 0.79 nA–0.43 nA-0.23 nA-80 pA and a voltage of 30 kV to minimize the taper effect and Ga^+^ damage. The ex situ micropillar compression tests (pillars with diameters of 1 and 3 μm) were performed on the nanoindenter equipped with a ∼5 μm diameter flat punch indenter (Hysitron TI Premier, Bruker, USA). This excludes the potential electron beam effect on the mechanical properties. All conditions in the ex situ micropillar compression tests repeated at least 3 times. The surface morphologies of the deformed micropillars were imaged by the scanning electron microscope (SEM, Apreo2 S Lovac, USA). The in situ nanopillar compression experiments were performed on the TEM (Thermo Fisher, Talos F200X G2, USA), equipped with a PI95 flat punch picoindenter (∼1 μm in diameter). All the compression modes were set to displacement-controlled, with a strain rate of ∼1 × 10^−3^ s^−1^.

### Bulk compression

For ambient temperature uniaxial bulk compression, (001) KTaO_3_ single-crystals with a dimension of 3 mm by 3 mm by 6 mm were acquired from Hefei Single Crystal Material Technology, Anhui, China. The long axis of the samples was aligned in the <001> direction. The surfaces of all the samples were polished to minimize surface damage. A constant strain rate of 1.5 × 10^−4^ s^−1^ was employed. The bulk samples were deformed on the uniaxial compression instrument (MTS E45, USA). The deformation images were collected in the VIC-gauge 2D software (Correlated Solution, Inc., USA). Before the compression testing, two Al_2_O_3_ plates were placed on top and bottom of the KTaO_3_ specimen to ensure smoother contact surfaces.

### Thermal conductivity measurement

The room temperature thermal conductivities of the SrTiO_3_ and KTaO_3_ single crystals with mechanically seeded dislocations were measured using the time-domain thermoreflectance (TDTR) method (*62*). Before the measurements, the mechanically seeded SrTiO_3_ and KTaO_3_ thin plates were coated with an aluminum (Al) layer with a thickness of ∼70 nm. All the thermal conductivity data of the mechanically seeded dislocation samples were collected in the middle of the wear tracks. The depth of the thermal conductivity measurement regions is around ∼300 nm. The objective lens is approximately 12 μm (1/e^2^ radius of 12 μm), and the relative modulation frequency is ∼10 MHz. Detailed data processing can be found in Reference (*33*).
