# Supplementary material for: Tunable dislocations overcome mechano-functional tradeoff in perovskite oxides
Source: Sci Adv. 2026 Aug 7;12(32):eaed0057. doi: 10.1126/sciadv.aed0057 (PMC13450299; doi:10.1126/sciadv.aed0057)
Supplement: Supplementary file 1 [file sciadv.aed0057_sm.pdf]

Science Advances
AAAS

Supplementary Materials for

Tunable dislocations overcome mechano-functional tradeoff in perovskite oxides

Jiawen Zhang *et al*.

Corresponding author: Wenjun Lu, luwj@sustech.edu.cn; Xufei Fang, xufei.fang@kit.edu

Sci. Adv. **12**, eaed0057 (2026)
DOI: 10.1126/sciadv.aed0057

The PDF file includes:

Figs. S1 to S16
Tables S1 and S2
Legends for movies S1 to S12

Other Supplementary Material for this manuscript includes the following:

Movies S1 to S12

Figs. S1 to S16

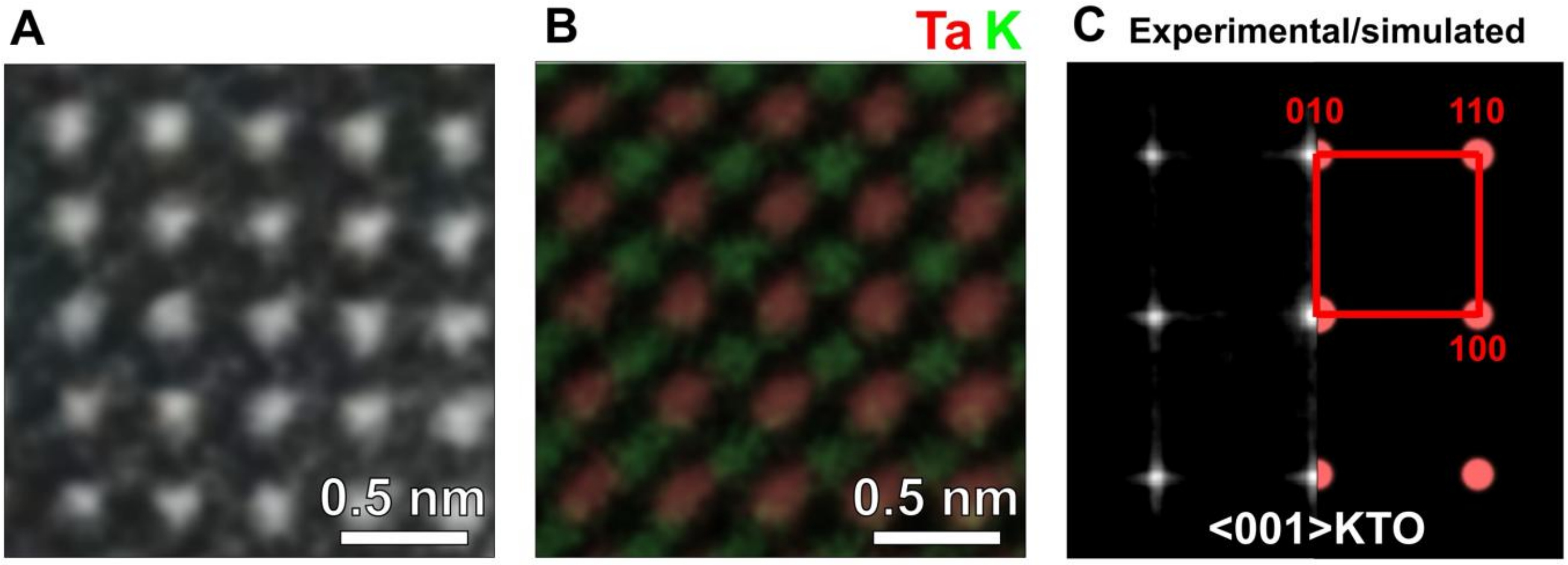

Fig. S1. Atomic-scale characterization of the KTO reference sample: (**A**) A HAADF-STEM image at atomic resolution. (**B**) Corresponding EDS elemental mapping of potassium (K) and tantalum (Ta). (**C**) Electron diffraction pattern with simulated data of KTO along the <001> zone axis.

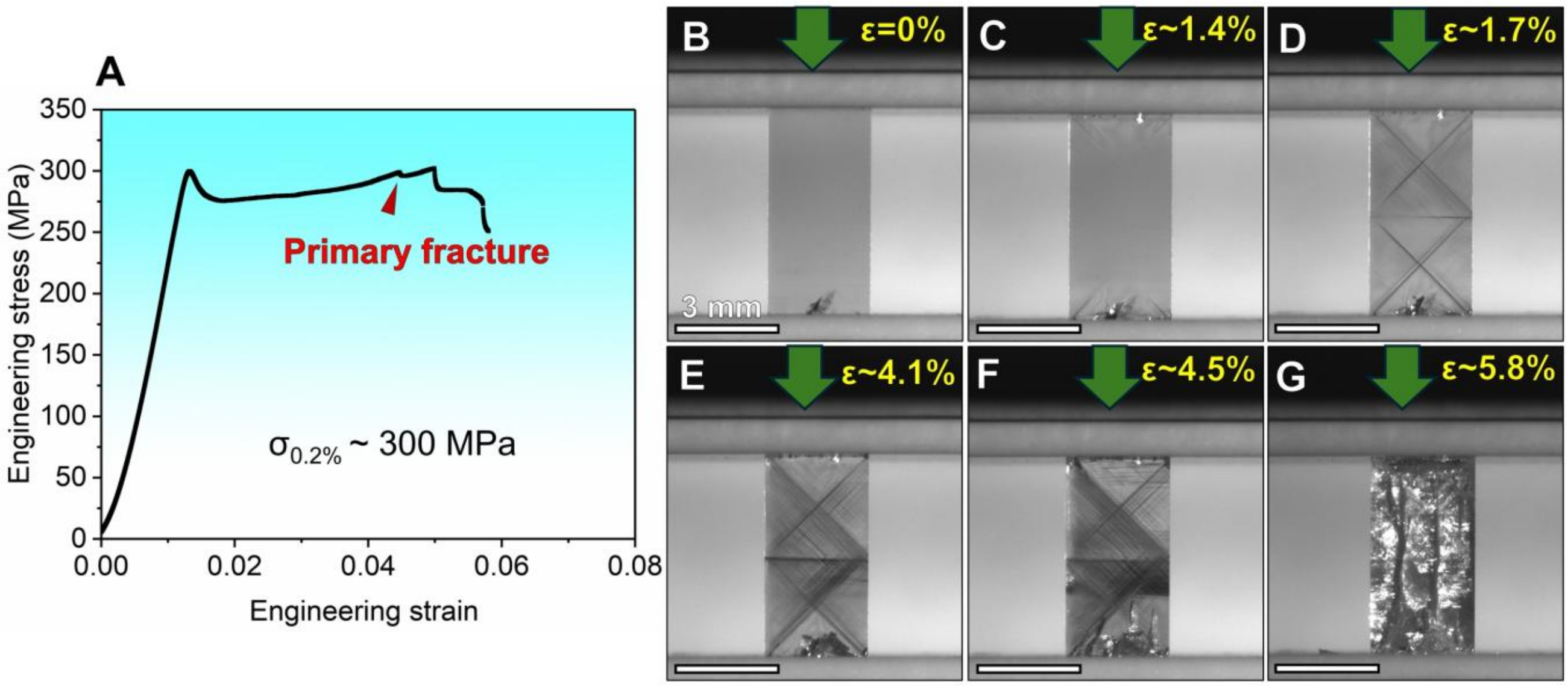

Fig. S2. Room-temperature bulk compression of single-crystal KTO along the <001> loading direction with a strain rate of ~10^{-4}/s. The slip traces (45-degree tilt dark lines with respect to the loading axis) indicate the <110> slip directions on the {110} slip planes. The sample is capable of ~5% plastic strain without critical fracture, and the lowest yielding point is around 270 MPa.

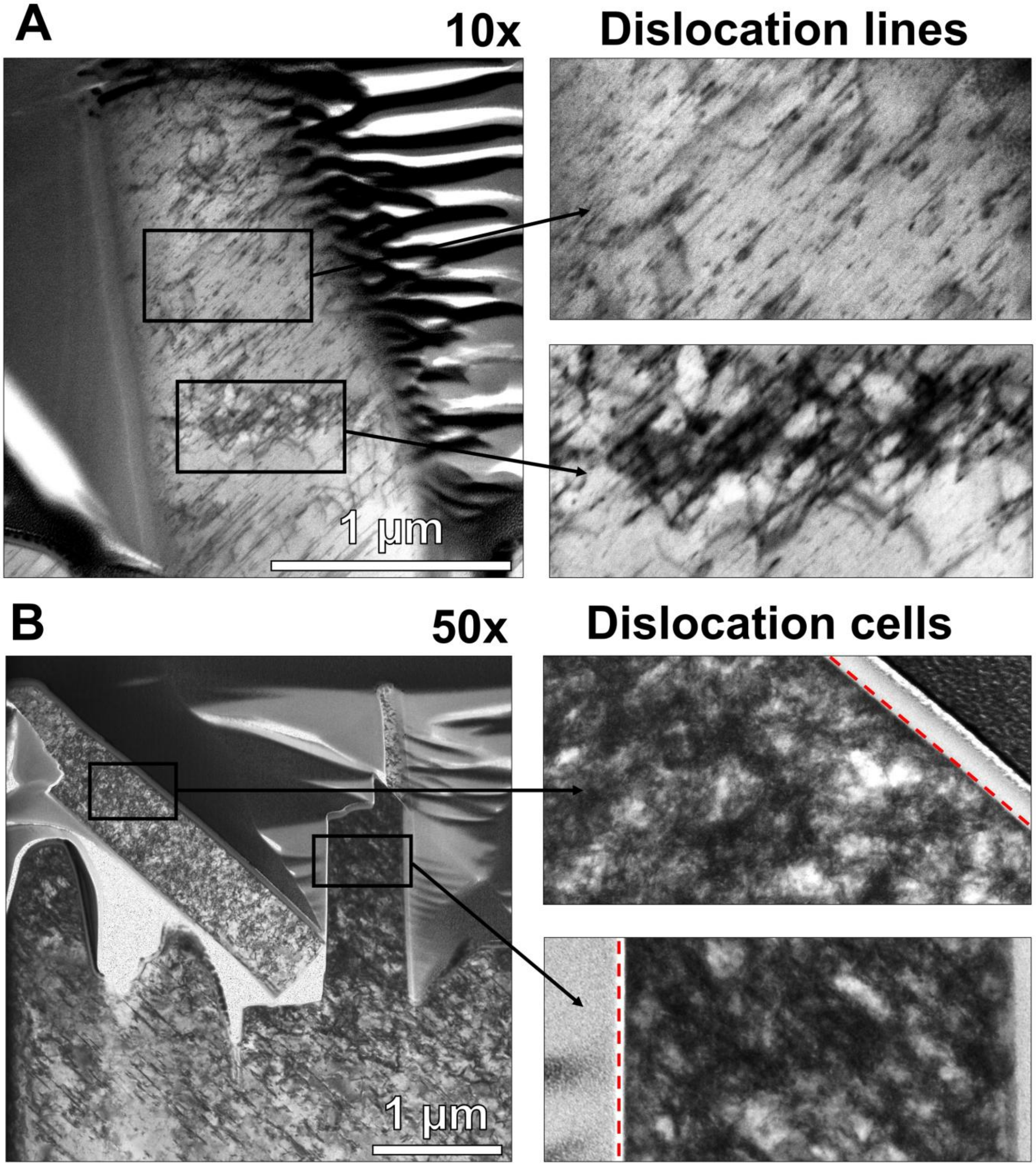

Fig. S3. Structural characterization of micropillars with two typical dislocation densities after compression: (**A**) An ABF-STEM image showing the morphology of the 10× scratched sample after compressive deformation up to 20% strain. (**B**) An ABF-STEM image displaying the fractured morphology of the 50× scratched sample after compressive deformation to 4.2% strain.

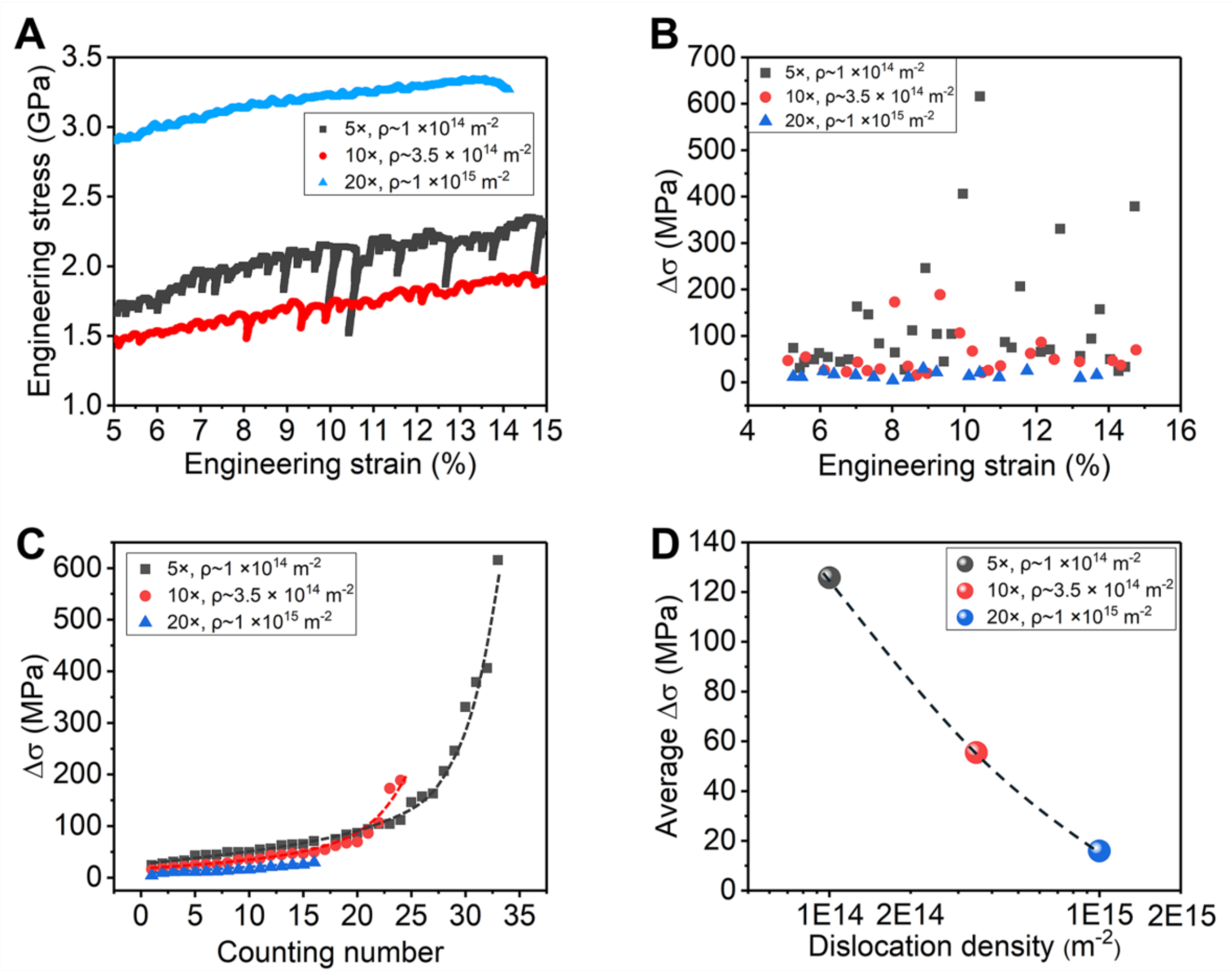

Fig. S4. Stress drop behavior of $KTaO_3$ micropillars (1 μm diameter) as a function of dislocation density. (A) Comparison of the stress-strain curves. (B) Calculated stress drop values (Δσ) versus strain. (C) Ranked Δσ values. (D) Average Δσ decreases with increasing dislocation density.

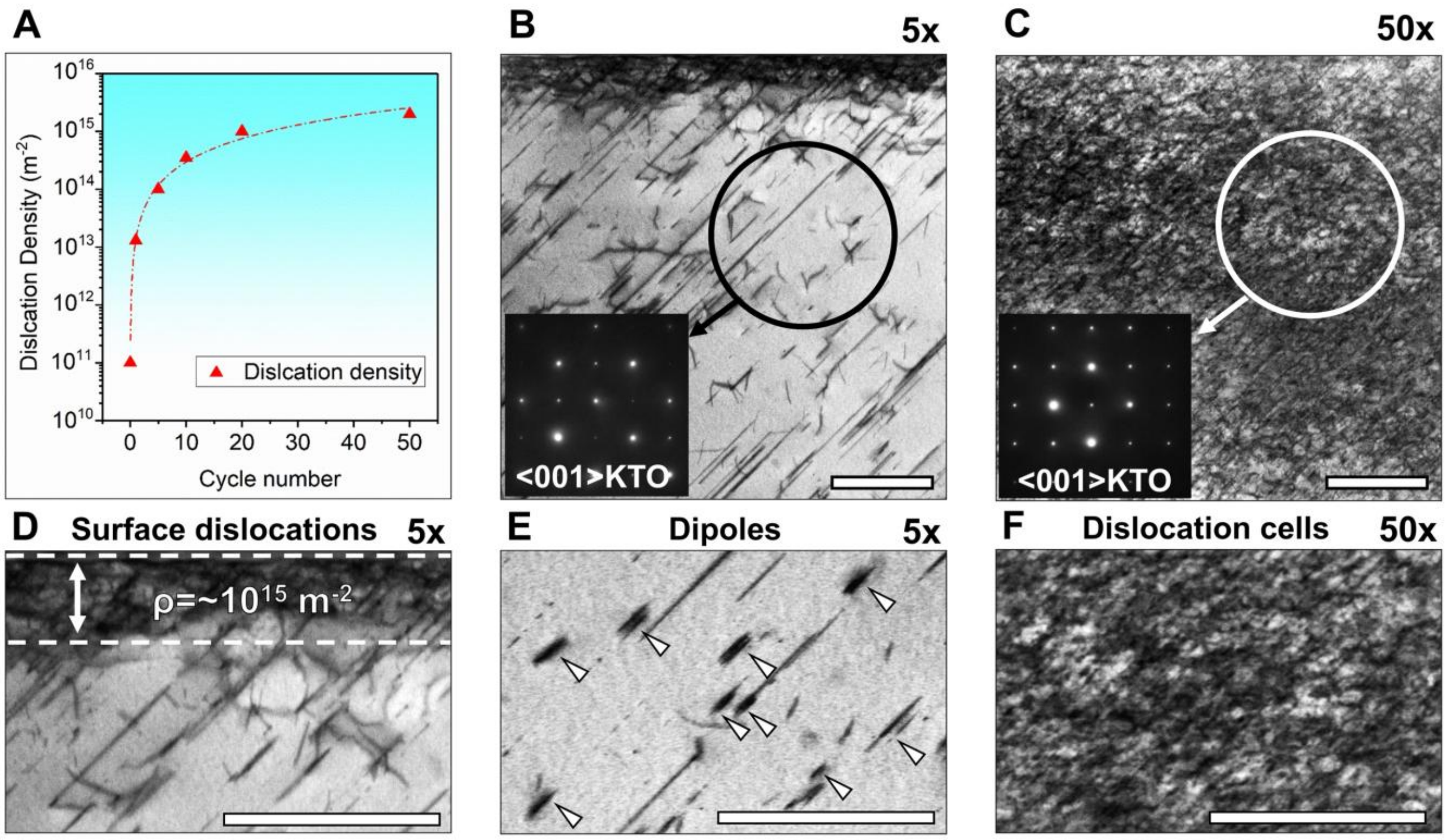

Fig. S5. An illustration of how repeated scratching progressively increases dislocation density and induces complex surface dislocation structures. (**A**) Relationship between dislocation density and the number of scratching cycles. (**B**) Low-magnification image of the 5× scratched sample. (**C**) Low-magnification image of the 50× scratched sample. (**D**) Magnified view of the 5× sample showing a gradient distribution of surface dislocations. (**E**) High-resolution image of the 5× sample revealing the presence of stacking faults or dislocation dipoles. (**F**) High-resolution image of the 5× sample illustrating the formation of dislocation cell structures at high dislocation density. The scale bars are 500 nm in all TEM images. Fig. S4 (B, C, and D) presents the same regions as in Fig. 2 (A_2 and A_5) in the main text, but at different magnifications to highlight the changes in dislocation density and structure.

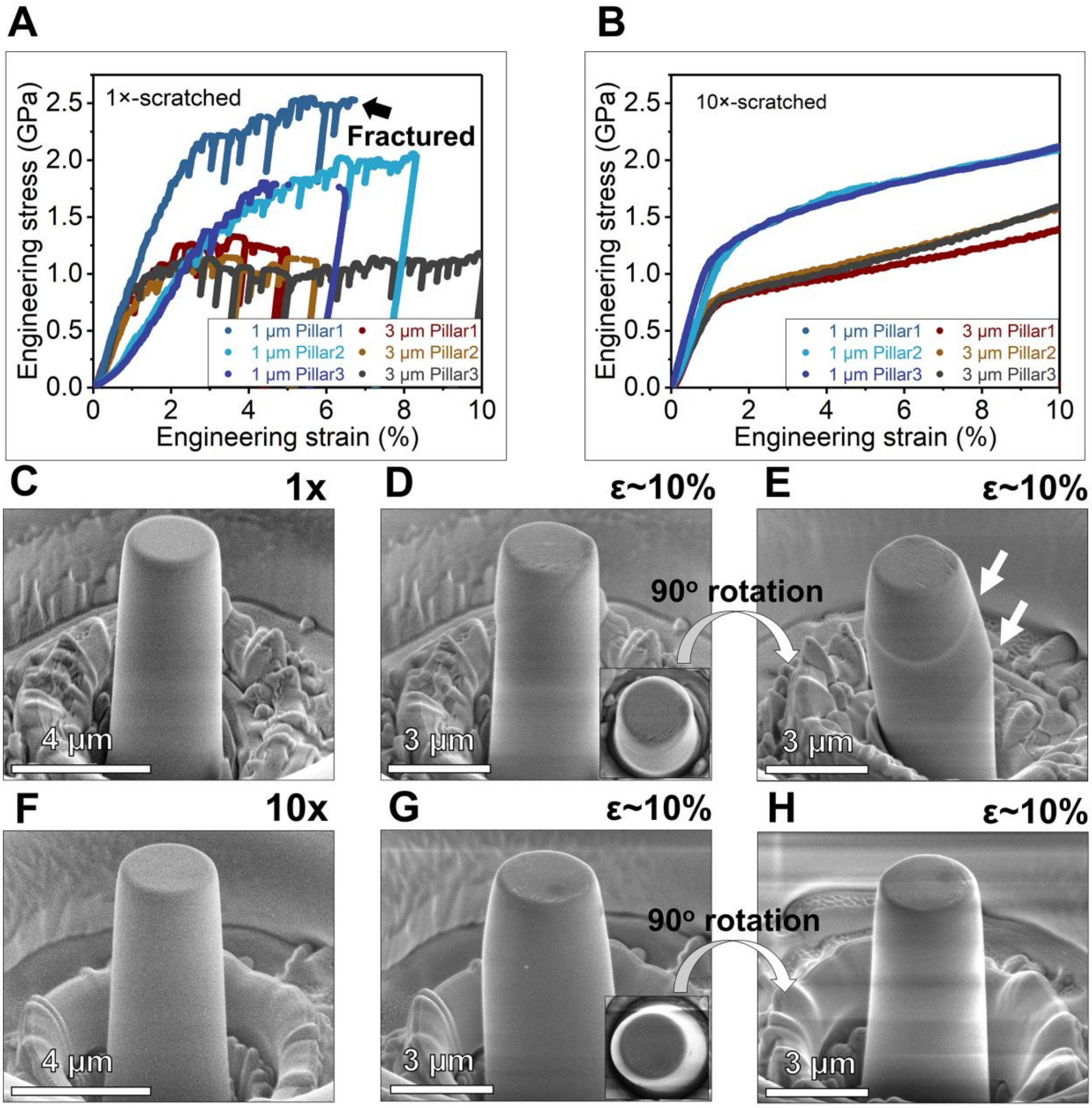

Fig. S6. Size effect mechanics and structural characterization of micropillar compression: (**A**) Engineering stress-strain curves of 1× scratched samples under size effect (pillar diameters: 1-3 μm). (**B**) Engineering stress-strain curves of 10× scratched samples under size effect (pillar diameters: 1-3 μm). (**C**) A SEM image of an undeformed 3 μm micropillar from the 1× sample. (**D**) A SEM image of the same pillar after 10% compressive strain. The inset is the top view of the pillar. (**E**) Side-view SEM image of the same pillar rotated by 90°, with arrows indicating visible slip bands. (**F**) SEM image of an undeformed 3 μm micropillar from the 10× sample. (**G**) SEM image of the 10× sample after 10% compressive strain. The inset is the top view of the pillar. (**H**) Side-view SEM image of the deformed pillar after 90° rotation.

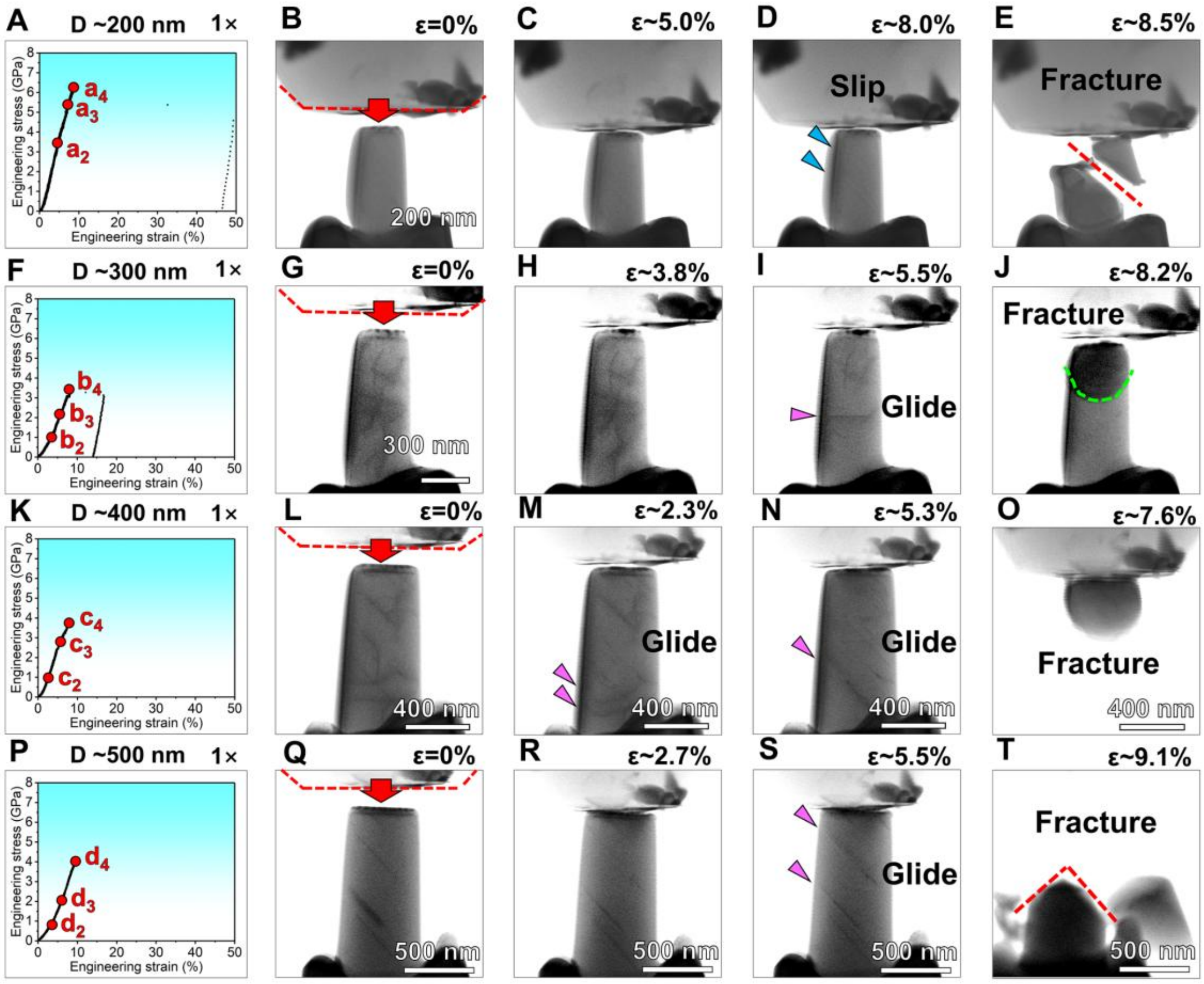

Fig. S7. Engineering stress-strain responses and in situ deformation behavior of 1× nanopillars under compression observed via ABF-STEM imaging: (**A**) Compressive stress–strain profile of the 1× sample with a diameter of 200 nm. (**B** to **E**) Sequential ABF-STEM images capturing the real-time deformation process of the 200 nm pillar. Fracture occurs at a maximum strain of approximately 8.5%. (**F**) Mechanical response of the 300 nm 1× nanopillar under uniaxial compression. (**G** to **J**) In situ ABF-STEM frames illustrating the deformation evolution. Dislocation glide (highlighted by the arrows) is clearly evident, with fracture taking place at around 8.2% strain. (**K**) Stress-strain behavior of the 400 nm 1× sample during compression. (**L** to **O**) Time-resolved ABF-STEM images revealing dislocation glide (purple arrow) and subsequent fracture at approximately 7.6% strain. (**P**) Load–displacement curve converted to engineering stress-strain for the 1× pillar with a 500 nm diameter. (**Q** to **T**) ABF-STEM snapshots tracking the deformation sequence. Dislocation motion (indicated by purple arrows) is distinctly observed, and fracture occurs at a strain level of about 9.1%. To facilitate a direct comparison of the deformation behavior of (001) $KTaO_3$ across different sample sizes, the same images in Fig. 3 (A to E) of the main text are reproduced in Fig. S7 (P to T).

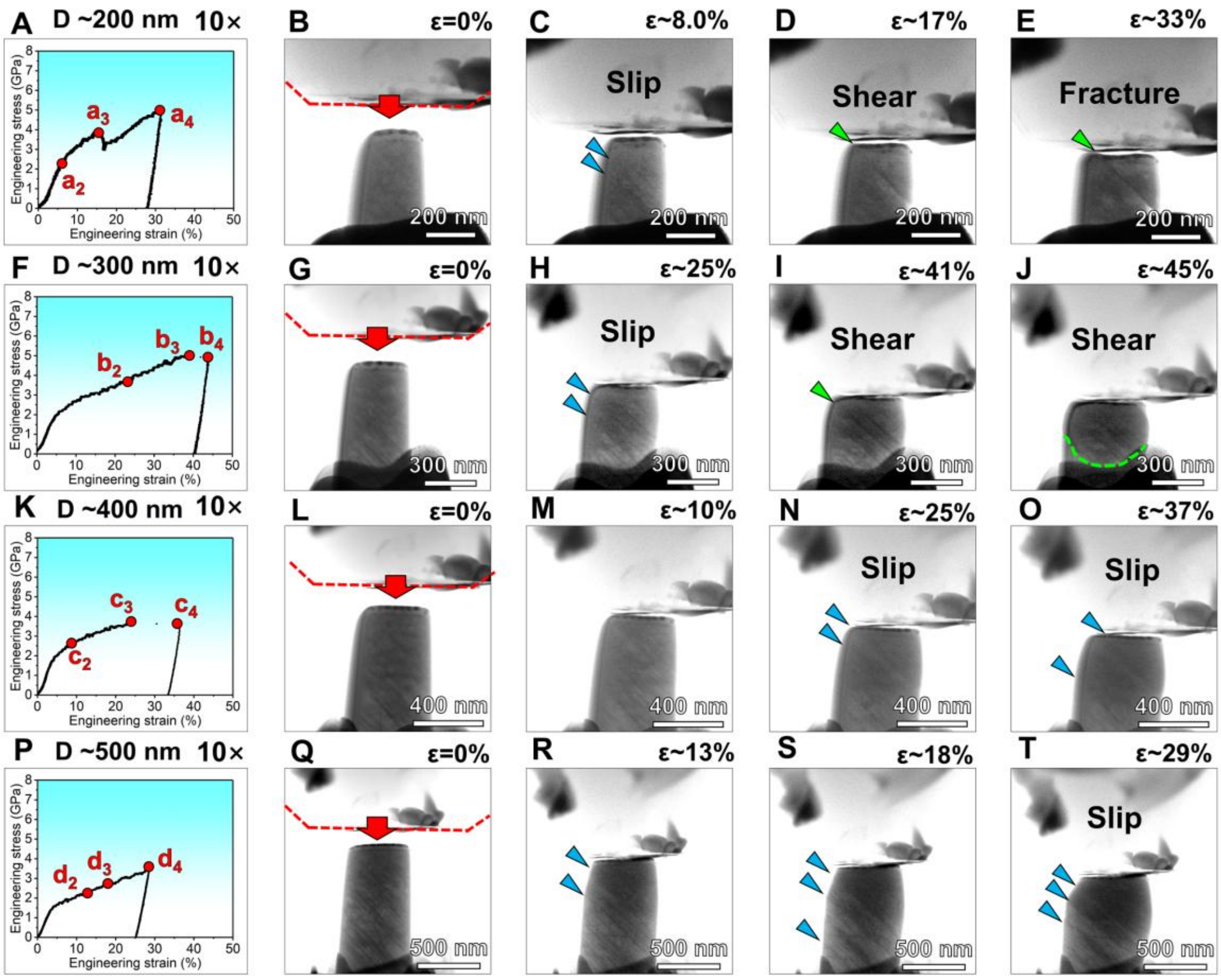

Fig. S8. Mechanical performance and in situ deformation characteristics of 10× nanopillars under compression, captured via ABF-STEM (dislocation slip and shear indicated by arrows): (**A**) Stress-strain curve of the 10× sample with a 200 nm diameter under compressive loading. (**B** to **E**) ABF-STEM image sequence documenting the deformation progression of the 200 nm pillar. Fracture occurs at an ultimate strain of approximately 33%. (**F**) Compressive behavior of the 300 nm 10× nanopillar. (**G** to **J**) In situ ABF-STEM frames detailing the deformation path, where both dislocation slip and shear activity are prominently observed. Shearing takes place at around 45% strain. (**K**) Mechanical response of the 400 nm sample during compression. (**L** to **O**) Time-lapse ABF-STEM images capturing the onset and development of dislocation glide, followed by plastic deformation without failure at roughly 37% strain. (**P**) Converted stress–strain profile from load-displacement data for the 500 nm pillar. (**Q** to **T**) ABF-STEM views revealing the dynamic deformation sequence, where intense dislocation slip (highlighted by arrows) precedes plastic deformation at a strain of approximately 29%. To facilitate a direct comparison of the deformation behavior of (001) $KTaO_3$ across different sample sizes, the same images in Fig. 3 (F to J) of the main text are reproduced in Fig. S8 (P to T).

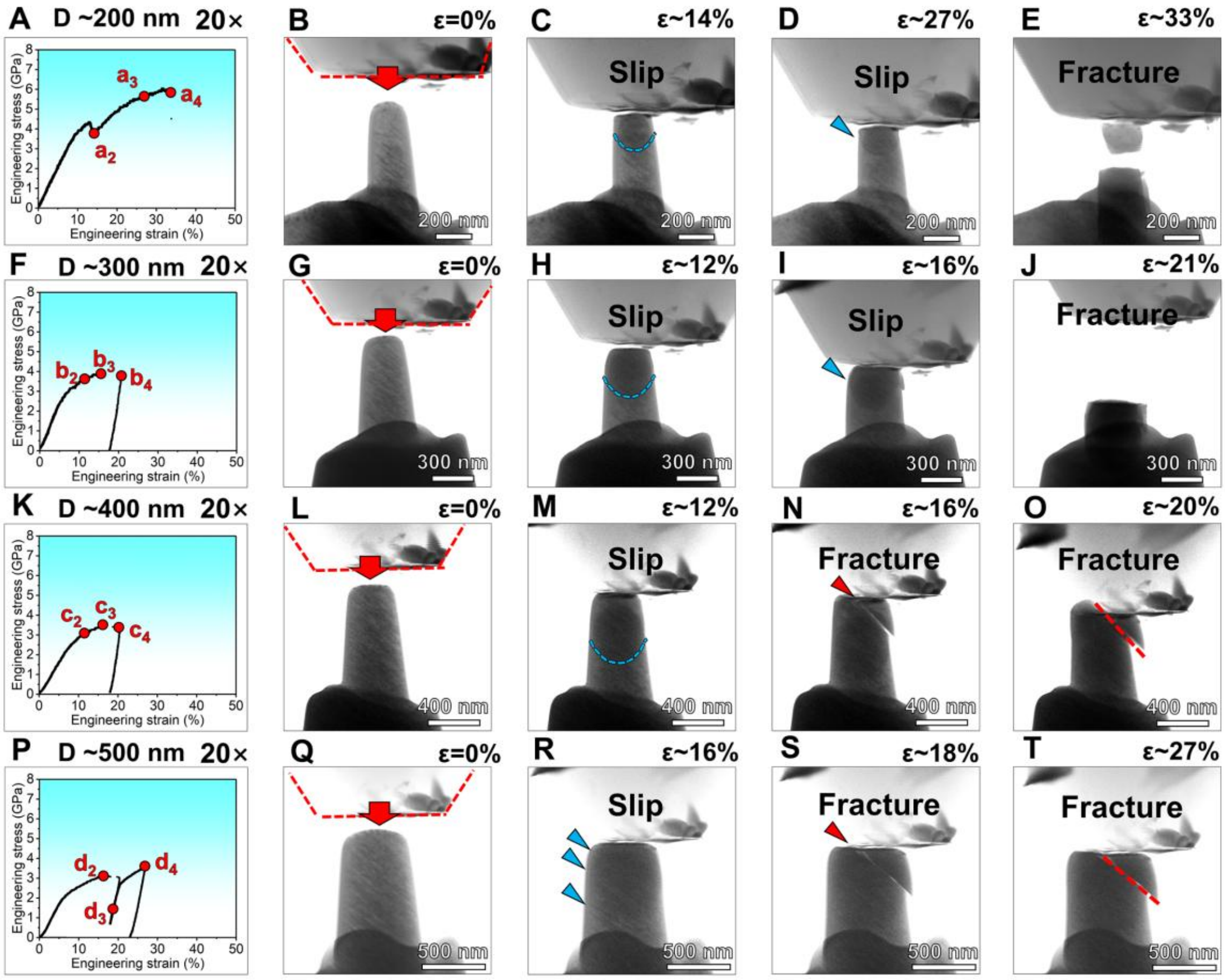

Fig. S9. Mechanical response and in situ deformation behavior of 20× nanopillars under compression, observed via ABF-STEM (dislocation slip and fracture denoted by arrows): (**A**) Compressive stress-strain profile of the 20× sample with a 200 nm diameter. (**B** to **E**) ABF-STEM series illustrating the progressive deformation of the 200 nm pillar, which eventually fractures at a strain of ~33%. (**F**) Deformation characteristics of the 300 nm nanopillar under axial loading. (**G** to **J**) Real-time ABF-STEM snapshots showing clear evidence of dislocation slip and shear activity, with fracture occurring around 21% strain. (**K**) Compressive performance of the 400 nm pillar. (**L** to **O**) Sequential STEM images capturing the initiation and propagation of dislocation motion (blue arrow), culminating in failure at approximately 20% strain. (**P**) Stress-strain relationship derived from load-displacement measurements for the 500 nm sample. (**Q** to **T**) STEM visualizations mapping the deformation evolution, where significant dislocation slip (marked in blue) precedes fracture at a strain close to 27%. To facilitate a direct comparison of the deformation behavior of (001) $KTaO_3$ across different sample sizes, the same images in Fig. 3 (K to O) of the main text are reproduced in Fig. S9 (P to T).

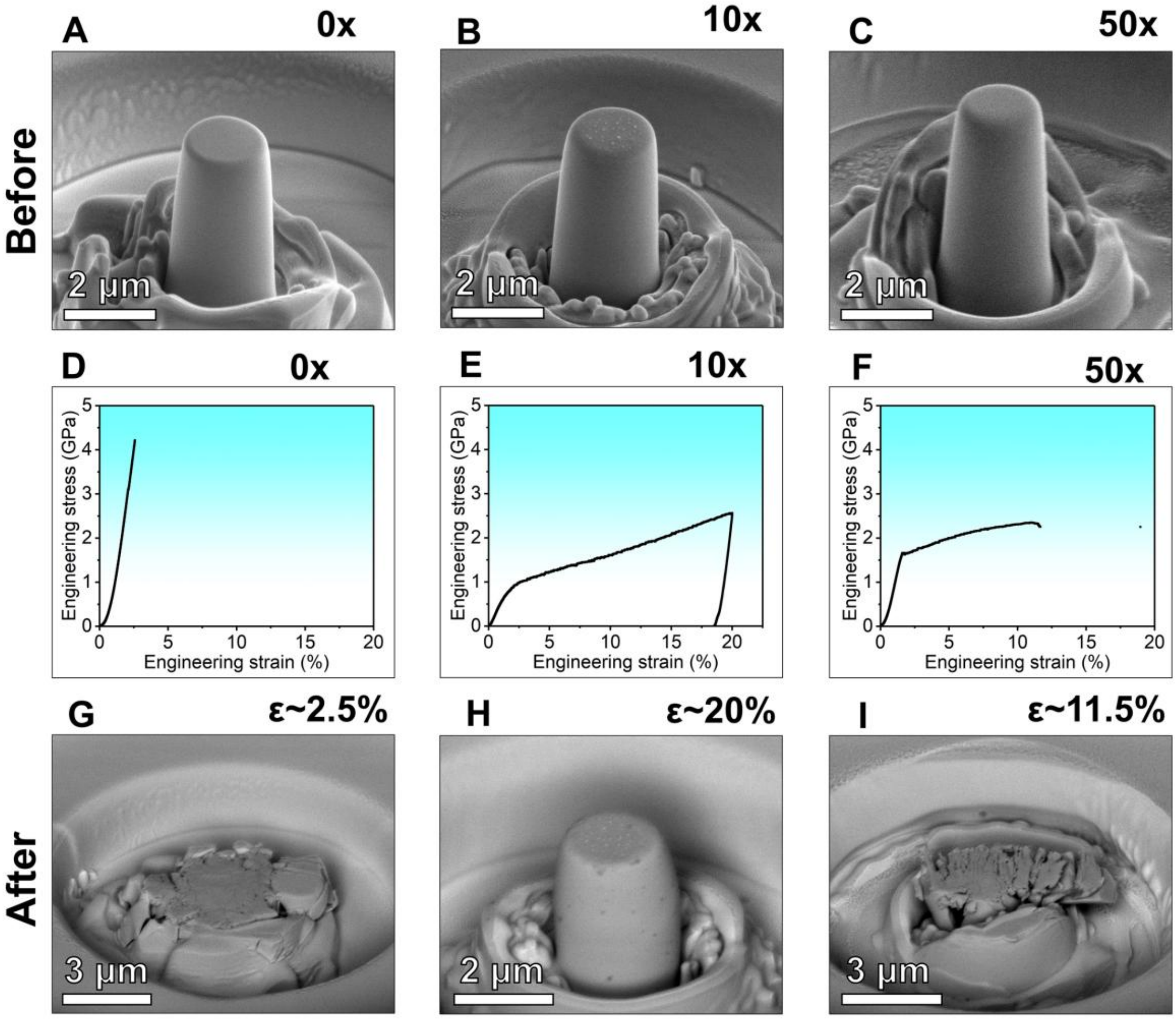

Fig. S10. Brittle-ductile-brittle transition of the KTO micropillar with a diameter of ~2 µm and different densities of mechanically seeded dislocations. (**A** to **C**) SEM images of the micropillars before deformation. (**D** to **F**) Engineering stress-strain curves of the deformed micropillars. (**G** to **I**) SEM images of the deformed micropillars, the reference sample in **G** shows completely brittle fracture, with dense dislocations in **H** displays plastic deformation, and the ultra-dense one in **I** again in a brittle manner.

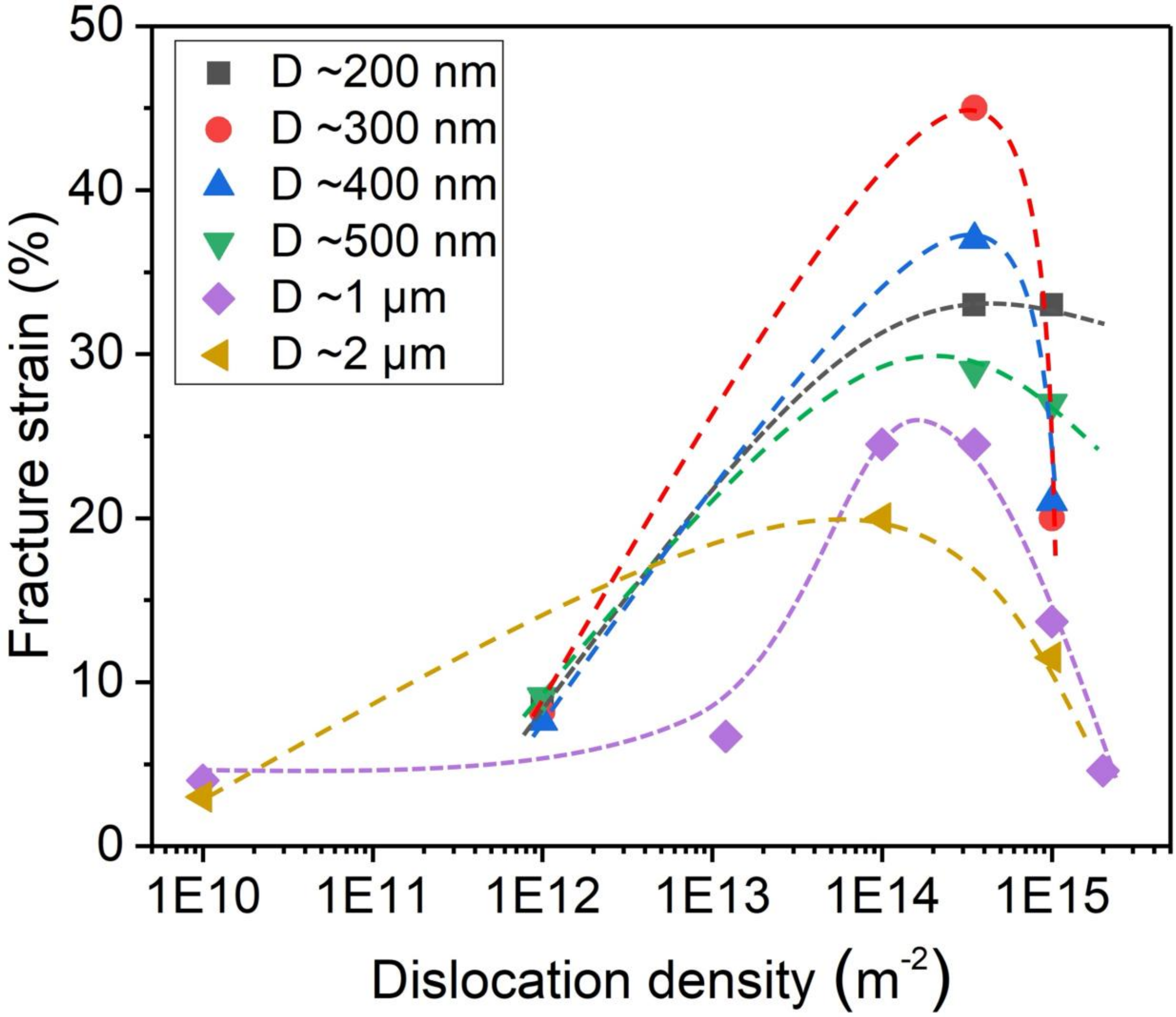

Fig. S11. Statistical trends in the fracture strain of the (001) $KTaO_3$ micropillar compression fractures with varying dislocation densities across different sizes.

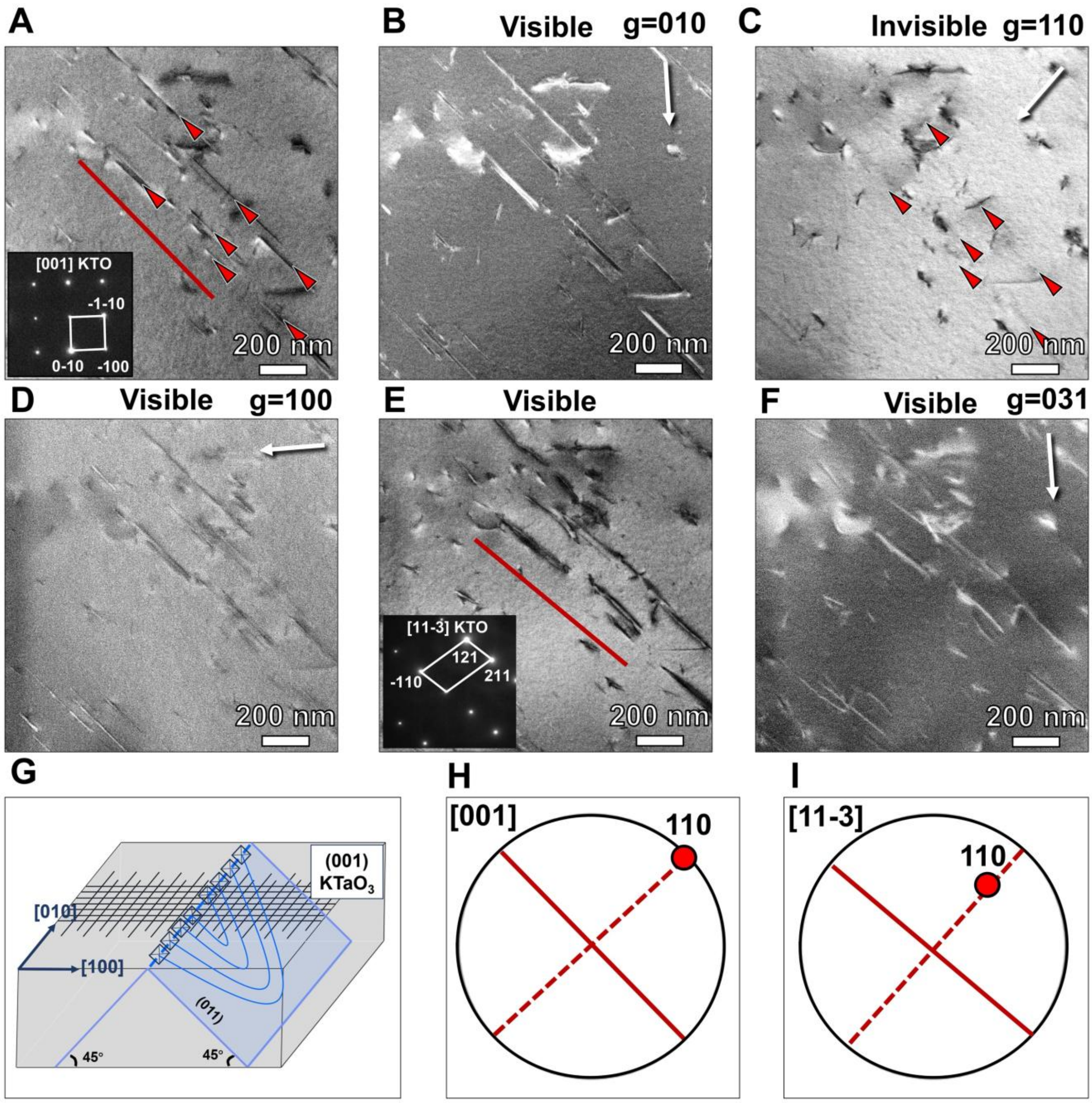

Fig. S12. Determination of the Burgers vector and slip system in the 10× scratched KTO sample: (**A**) Dislocation structure of single-crystal KTO along the <001> zone axis. The arrows indicate a dislocation line oriented at 45°, with the slip plane identified as {110}. (**B**) Under a diffraction vector ***g*** = 010, the dislocation line remains visible (no extinction). (**C**) Under ***g*** = 110, the dislocation line is extinguished. (**D**) Under ***g*** = $\bar{1}\bar{1}0$, the dislocation line remains visible (no extinction). (**E**) Dislocation structure of single-crystal KTO along the <11$\bar{3}$> zone axis. (**F**) Under ***g*** = 031, the dislocation line remains visible (no extinction). (**G**) Schematic illustration of the activated slip systems in single-crystal KTO. (**H**) Pole figure of single-crystal KTO along the <001> axis, where poles indicate edge-on slip

planes. (**I**) Pole figure of single-crystal KTO along the $<11\bar{3}>$ axis, where poles represent inclined slip planes.

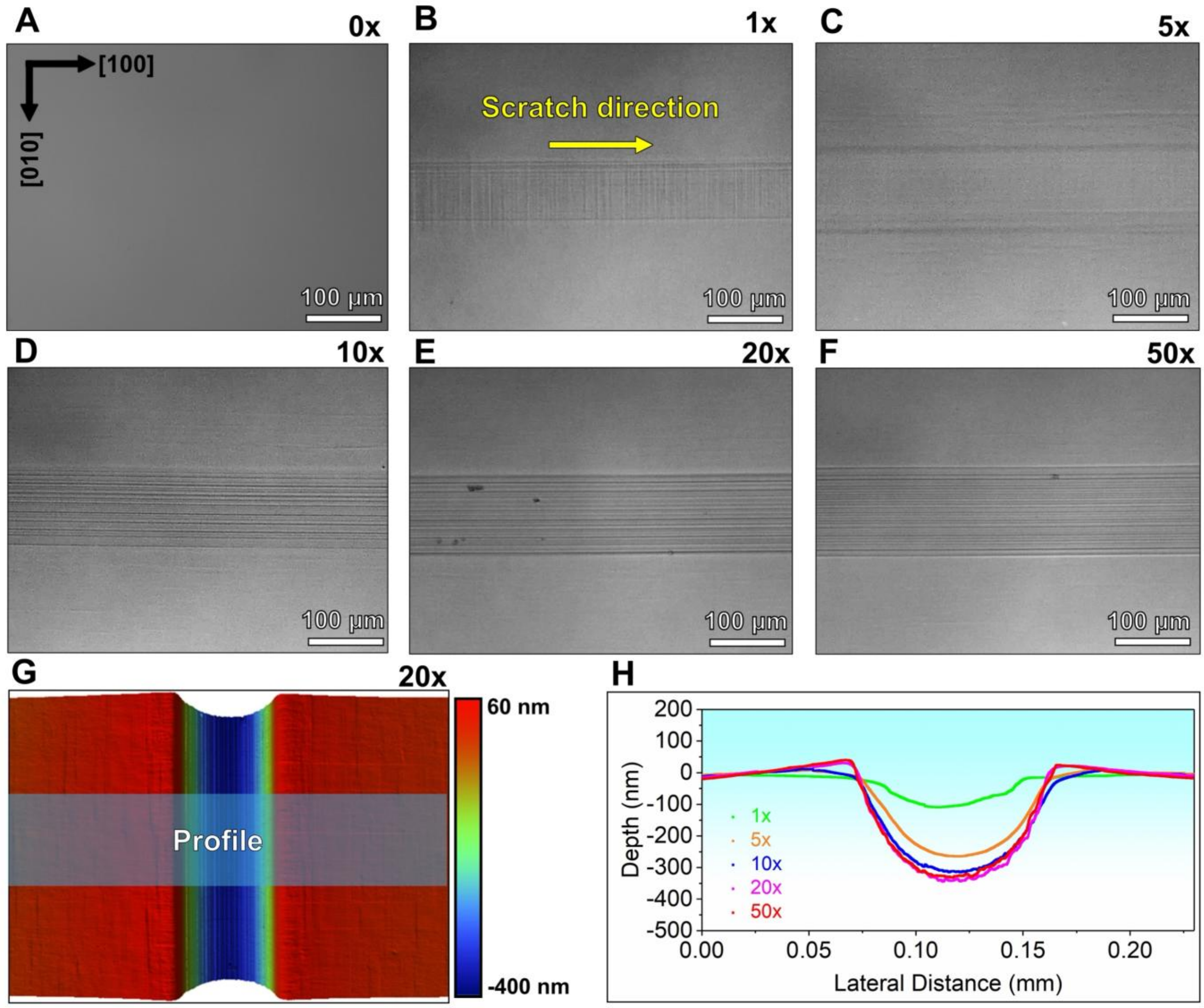

Fig. S13. Optical microscopy images of KTO samples subjected to different numbers of scratching cycles: (**A**) 0×; (**B**) 1×; (**C**) 5×; (**D**) 10×; (**E**) 20×; and (**F**) 50×. (**G**) Surface topography of the 20× scratched sample obtained by white-light interferometry. (**H**) Depth profiles of surface indentations along the Z-axis for samples with varying scratch cycles.

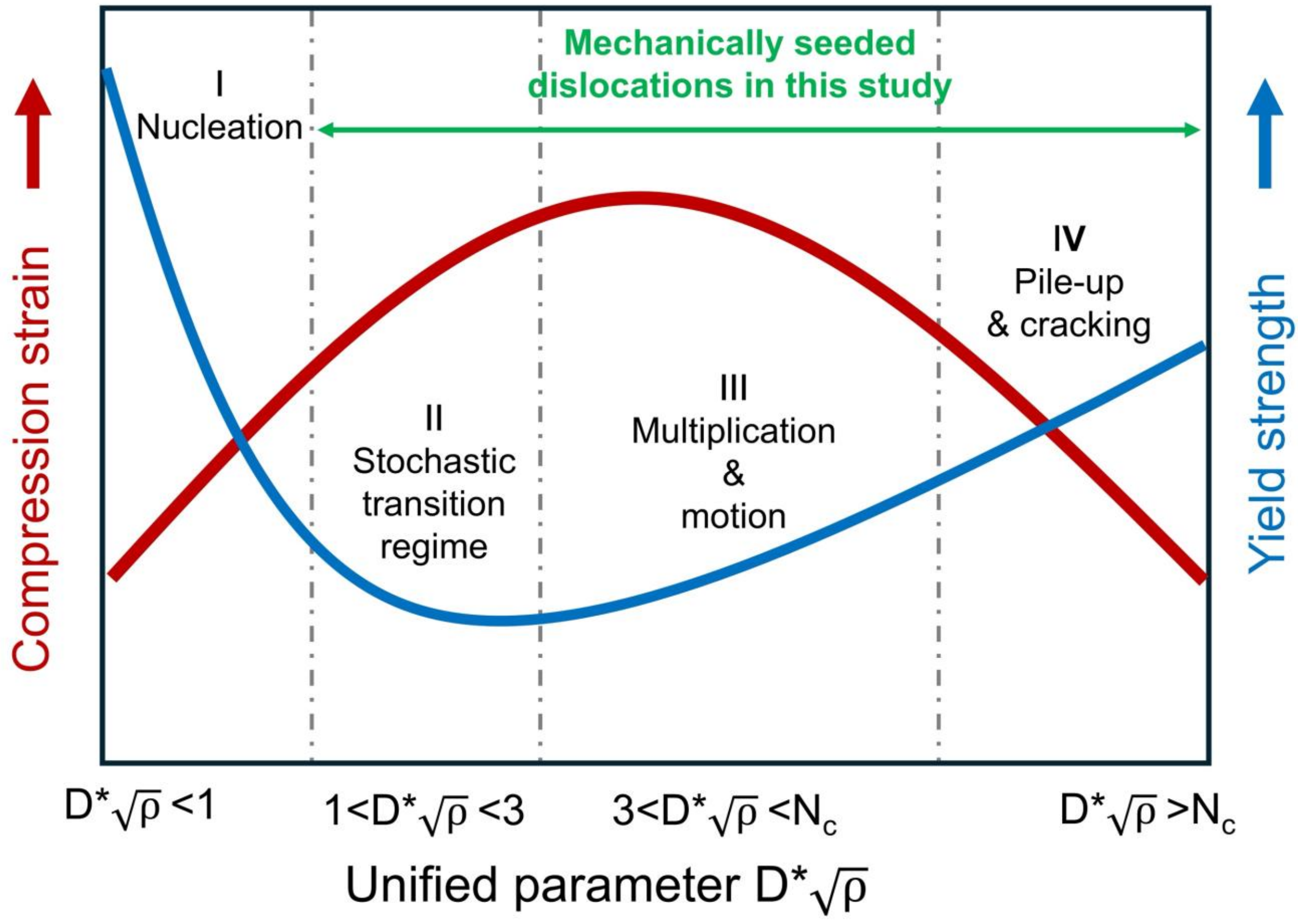

Fig. S14. **Compression fracture strain and yield strength as a function of the unified parameter $D^*\sqrt{\rho}$. N_c indicates a critical number of dislocations in the deformed volume.**

To highlight the dislocation structures within the pillar for the main Figure, we have adjusted the brightness and contrast of the original STEM image. The original, unprocessed STEM image below (**Fig. S15**). In these raw images, it is clear that the flat punch indenter is properly aligned and in full, uniform contact with the top surface of the micropillar prior to fracture.

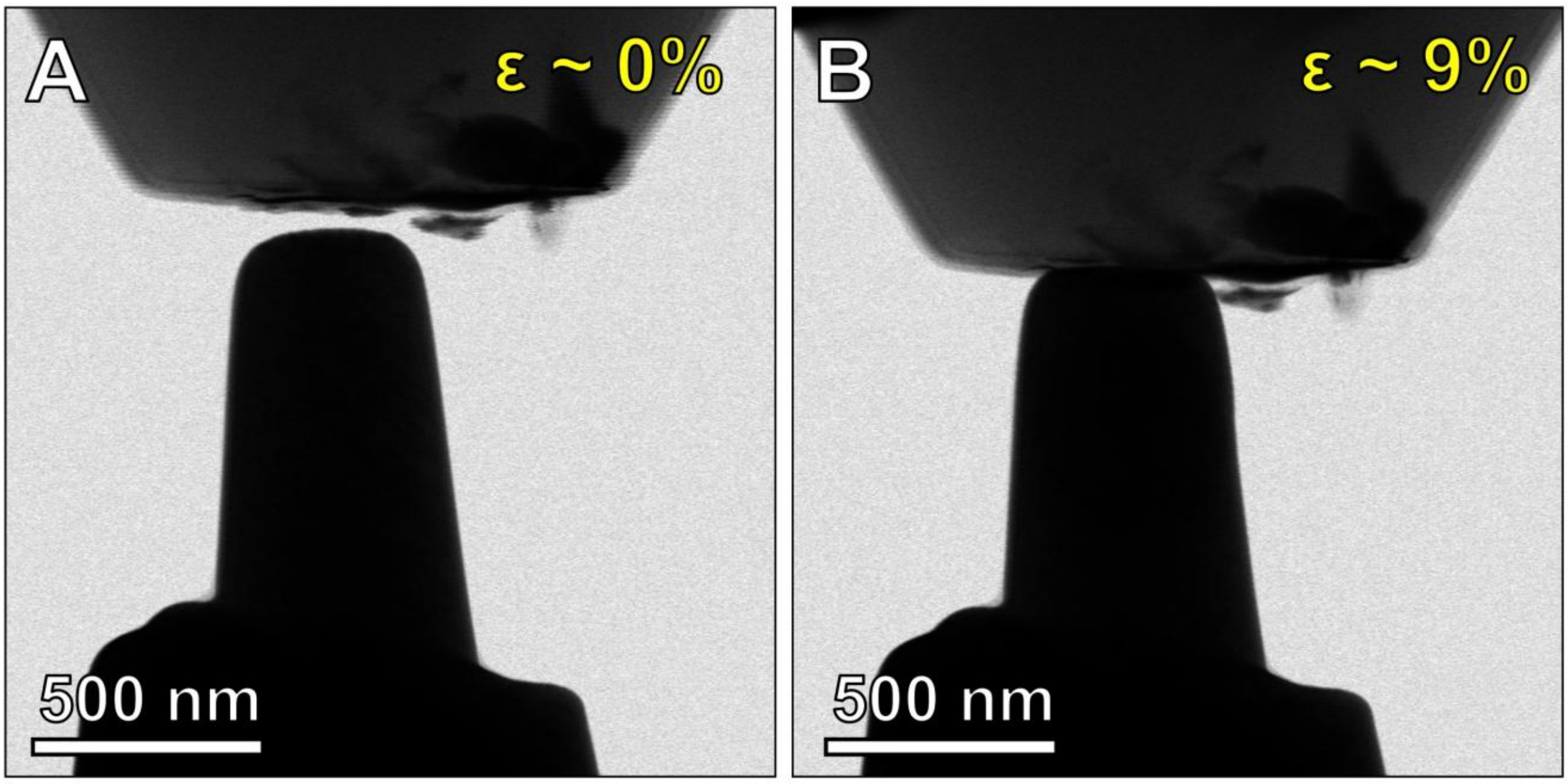

Fig. S15. The original STEM images illustrate the position between the indenter and the pillar in Fig. 3C in the manuscript.

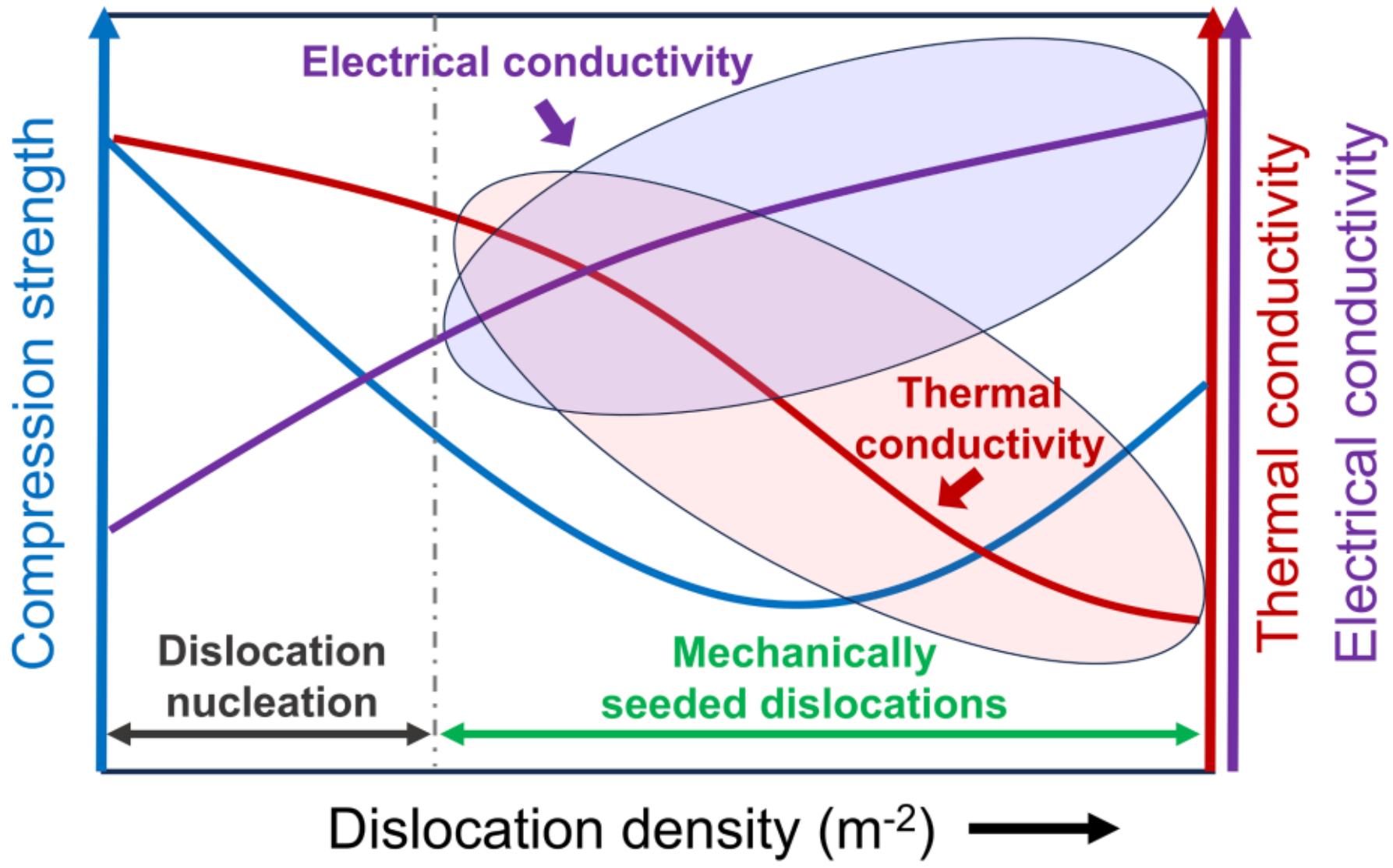

Fig. S16. Predicted general relationship between the dislocation density and functionality of the mechanically seeded dislocations perovskite oxide ceramics, taking $SrTiO_3$ as an example.

Tables S1 to S2

Table S1. Summary of the compression mechanical properties of the (001) $KTaO_3$ with different dislocation densities and a diameter of 1 μm.

Scratching passes	Dislocation density (m^{-2})	Yield strength ($\sigma_{0.2}$, GPa)	Fracture strength (σf, GPa)	Fracture stain (%)
0×	~10^{10} - 10^{11}	/	7.5 ± 0.2	3.5 ± 0.3%
1×	~10^{13}	1.4 ± 0.2	1.6 ± 0.0	6.5% ± 1.2%
5×	~10^{14}	0.7 ± 0.1	2.2 ± 0.0	24.5% ± 0.5%
10×	~3.5 × 10^{14}	1.0 ± 0.1	2.9 ± 0.1	24.5 ± 0.2%
20×	~10^{15}	2.0 ± 0.2	3.0 ± 0.2	13.7 ± 0.4%
50×	~2 × 10^{15}	1.8 ± 0.1	2.5 ± 0.1	4.6 ± 0.9%

Note: The reference sample (0×) exhibits only elastic deformation and therefore has no yield strength.

Table S2. Calculated results of the dislocation spacing of the (001) $KTaO_3$ crystal with different dislocation densities in this study.

	Dislocation densities				
	10^{10} m^{-2}	1.2×10^{13} m^{-2}	3.5×10^{14} m^{-2}	1×10^{15} m^{-2}	2×10^{15} m^{-2}
$1/\sqrt{\rho}$	10 µm	300 nm	50 nm	30 nm	20 nm
$D^*\sqrt{\rho}$, D ~ 200 nm	0.02, NA	0.07, X	4, O	7, O	10, NA
$D^*\sqrt{\rho}$, D ~ 300 nm	0.03, NA	0.1, X	6, O	10, O	15, NA
$D^*\sqrt{\rho}$, D ~ 400 nm	0.04, NA	1.3, X	8, O	13, O	20, NA
$D^*\sqrt{\rho}$, D ~ 500 nm	0.05, NA	1.7, X	10, O	17, O	25, NA
$D^*\sqrt{\rho}$, D ~ 1µm	0.1, X	3, X and O	20, O	30, O	50, X
$D^*\sqrt{\rho}$, D ~ 2µm	0.2, X	6, NA	40, O	60, X	100, X

Note: X represents brittle, O represents plastic deformation, and NA represents not available.

Legends for movies S1 to S12

Please see the uploaded Movies:

Movie S1. In situ TEM Movie of nanopillar compression of the (001) KTO with low dislocation density (1× scratching) and a diameter of ~200 nm.

Movie S2. In situ TEM Movie of nanopillar compression of the (001) KTO with low dislocation density (1× scratching) and a diameter of ~300 nm.

Movie S3. In situ TEM Movie of nanopillar compression of the (001) KTO with low dislocation density (1× scratching) and a diameter of ~400 nm.

Movie S4. In situ TEM Movie of nanopillar compression of the (001) KTO with low dislocation density (1× scratching) and a diameter of ~500 nm.

Movie S5. In situ TEM Movie of nanopillar compression of the (001) KTO with medium dislocation density (10× scratching) and a diameter of ~200 nm.

Movie S6. In situ TEM Movie of nanopillar compression of the (001) KTO with medium dislocation density (10× scratching) and a diameter of ~300 nm.

Movie S7. In situ TEM Movie of nanopillar compression of the (001) KTO with medium dislocation density (10× scratching) and a diameter of ~400 nm.

Movie S8. In situ TEM Movie of nanopillar compression of the (001) KTO with medium dislocation density (10× scratching) and a diameter of ~500 nm.

Movie S9. In situ TEM Movie of nanopillar compression of the (001) KTO with high dislocation density (20× scratching) and a diameter of ~200 nm.

Movie S10. In situ TEM Movie of nanopillar compression of the (001) KTO with high dislocation density (20× scratching) and a diameter of ~300 nm.

Movie S11. In situ TEM Movie of nanopillar compression of the (001) KTO with high dislocation density (20× scratching) and a diameter of ~400 nm.

Movie S12. In situ TEM Movie of nanopillar compression of the (001) KTO with high dislocation density (20× scratching) and a diameter of ~500 nm.
